# Phase Separation of TRIM21 Modulates PTPN14 Stability to Drive Flow‐Dependent Endothelial Activation and Atherogenesis

**DOI:** 10.1002/advs.77262

**Published:** 2026-08-18

**Authors:** Xue He, Qiannan Ma, Xue Wang, Jiaxu Mang, Chufeng Li, Bingjun Wu, Liu Yao, Yequn Chen, Qiankun Bao, Yi Zhu, Jinlong He

**Affiliations:** ^1^ Province and Ministry Co‐Sponsored Collaborative Innovation Center for Medical Epigenetics Eye Institute and School of Optometry Tianjin Medical University Eye Hospital Tianjin Medical University Tianjin China; ^2^ NHC Key Laboratory of Hormones and Development Department of Physiology and Pathophysiology Tianjin Medical University Tianjin China; ^3^ Department of Cardiology First Affiliated Hospital of Shantou University Medical College Guangdong China; ^4^ Tianjin Key Laboratory of Ionic‐Molecular Function of Cardiovascular Disease Department of Cardiology Tianjin Institute of Cardiology the Second Hospital of Tianjin Medical University Tianjin China

**Keywords:** atherosclerosis, endothelial mechanotransduction, PTPN14, shear stress, TRIM21

## Abstract

Local flow patterns determine the uneven distribution of atherosclerotic lesions, yet the underlying mechanism remains poorly defined. We previously reported that protein tyrosine phosphatase nonreceptor type 14 (PTPN14) is implicated in atherosclerosis. Its role in flow‐dependent endothelial dysfunction and the upstream regulatory mechanisms remain incompletely understood. Here, we show that PTPN14 functions as a flow‐sensitive regulator in endothelial cells, with its protein stability reduced under disturbed flow. Endothelial‐specific deletion of PTPN14 led to exacerbated endothelial inflammation and atherosclerosis in vivo. We further identify TRIM21 as a critical E3 ubiquitin ligase that binds PTPN14 via its SPRY domain and promotes K48‐linked polyubiquitination at lysine 956, resulting in proteasomal degradation of PTPN14. Notably, disturbed flow induced TRIM21 LLPS in a cytoskeleton‐dependent manner, facilitating the recruitment of PTPN14 into co‐condensates and enhancing its ubiquitination. Endothelial‐specific TRIM21 overexpression aggravated disturbed flow‐induced endothelial inflammation and atherosclerosis. In contrast, endothelial‐specific TRIM21 deficiency attenuated induced endothelial activation and atherosclerosis; importantly, this atheroprotective effect was abolished by concomitant PTPN14 deletion, establishing a functional TRIM21‐PTPN14 regulatory axis in vivo. Together, these findings uncover phase separation‐dependent ubiquitin signaling as a mechanistic link between hemodynamic forces and endothelial dysfunction and suggest the TRIM21‐PTPN14 pathway as a potential therapeutic target in atherosclerosis.

## Graphical abstract

Disturbed flow promotes the formation of TRIM21‐rich biomolecular droplets, which concentrate TRIM21 and PTPN14 and facilitate their SPRY‐FERM interaction (illustrated by the TRIM21 D355‐PTPN14 R132 salt bridge). This condensate‐driven proximity enables TRIM21 to catalyze K48‐linked polyubiquitination of PTPN14 at lysine 956, leading to proteasome‐dependent degradation, endothelial activation, and an athero‐susceptible phenotype. In contrast, athero‐protective flow promotes dispersal of TRIM21 condensates, thereby limiting TRIM21‐PTPN14 colocalization, preserving PTPN14 protein stability, and maintaining endothelial homeostasis and athero‐resistance.

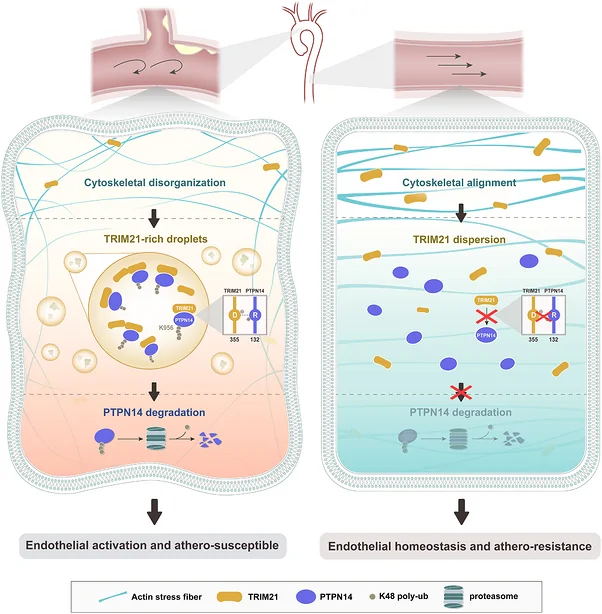

## 1 Introduction

Atherosclerosis leads to the cause of mortality and morbidity worldwide. Atherosclerotic plaques generally accumulate over many years and ultimately present clinically as ischemic injury, including myocardial infarction, stroke, or peripheral arterial disease. In addition to acute manifestations, atherosclerosis is increasingly recognized as a key contributor to long‐term complications such as heart failure and chronic kidney dysfunction [1]. Despite substantial advances in lipid‐lowering treatments, including statins and PCSK9 inhibitors, which have effectively decreased cardiovascular mortality over recent decades, the burden of coronary artery disease has continued to rise in the past ten years [2], highlighting an urgent need for novel therapeutic strategies for atherosclerosis beyond lipid‐lowering approaches.

Endothelial cells (ECs) lining the arterial lumen are continuously exposed to shear stress generated by blood flow, a critical determinant of endothelial homeostasis that governs both the progression and focal distribution of atherosclerotic plaques [3]. Hemodynamic forces act on ECs by reshaping cellular morphology and the cytoskeletal architecture, modulating intracellular signaling pathways, and reprogramming gene expression, thereby regulating endothelial phenotype and function [4]. Endothelial dysfunction preferentially develops at arterial branches and curvatures, where blood flow is disturbed. In contrast, pulsatile shear stress (PSS) present in straight regions of the arterial tree exerts atheroprotective effects [5]. Elucidating the molecular mechanisms by which altered hemodynamic forces regulate endothelial function is therefore essential for identifying novel therapeutic targets for atherosclerosis.

ECs sense shear stress through a repertoire of mechanosensors localized to the apical surface, which convert mechanical cues from blood flow into intracellular signaling events and adaptive cellular behaviors. Accumulating evidence has highlighted a pivotal role for Yes‐associated protein (YAP), a transcriptional co‐activator downstream of the Hippo pathway, in endothelial mechanotransduction and phenotypic regulation. Both our group and others have demonstrated that YAP subcellular localization is tightly regulated by distinct flow patterns. Exposure of ECs to disturbed flow promotes YAP activation, thereby driving pro‐atherogenic endothelial phenotypes and accelerating atherosclerotic lesion formation [6, 7, 8]. Protein tyrosine phosphatase non‐receptor type 14 (PTPN14), a critical negative regulator of YAP signaling, encodes a highly conserved tyrosine phosphatase originally identified in a screen for phosphatases expressed in normal human breast tissue [9]. PTPN14 contains two PPxY motifs that facilitate the recruitment of large tumor suppressor kinases 1/2 (LATS1/2) and YAP, thereby acting as a scaffold to promote LATS1/2‐dependent phosphorylation and inactivation of YAP [10]. Owing to its ability to restrain YAP signaling, PTPN14 functions as a putative tumor suppressor [10, 11, 12]. We previously reported that PTPN14 interacts with YAP in ECs, as identified by LC‐MS analysis [13]. In addition, we found that Harmine, a natural compound that could inhibit disturbed flow‐induced YAP activation via upregulating PTPN14 expression in ECs, exerts atheroprotective effects in both *ApoE^−/−^* and *Ldlr^−/−^* mouse models [14]. Despite these observations implicating endothelial PTPN14 as a critical regulator of atherosclerosis, its regulation by distinct flow patterns and its mechanistic role in atherogenesis remain poorly defined.

In the present study, we demonstrate that PTPN14 protein expression is differentially regulated by distinct flow patterns both in vivo and in vitro. We further identify tripartite motif‐containing protein 21 (TRIM21), a previously underexplored E3 ubiquitin ligase in ECs, as a critical regulator of PTPN14 stability. By constructing endothelial‐specific PTPN14, TRIM21, and double‐knockout mouse models, we evaluated their roles in atherosclerosis development. Mechanistically, we demonstrate that TRIM21 undergoes liquid–liquid phase separation (LLPS), particularly under disturbed flow conditions, which facilitates PTPN14 ubiquitination and degradation. Collectively, our findings establish the TRIM21‐PTPN14 axis as a hemodynamics‐sensitive regulatory pathway and a potential therapeutic target for atherosclerosis.

## 2 Results

### 2.1 PTPN14 Protein Levels and Stability are Regulated By Distinct Flow Patterns

Given the spatial heterogeneity of atherosclerotic plaque distribution, where lesions preferentially form at arterial bifurcations and curvatures exposed to low and/or oscillatory (disturbed) shear stress [15], we first examined PTPN14 expression in aortic intima of *ApoE^−/−^* mice. PTPN14 expression in endothelium was markedly higher in the thoracic aorta (TA), a region exposed to high, unidirectional laminar shear stress, compared with the aortic arch (AA), which experiences low and oscillatory shear stress (Figure 1A–C). Quantitative PCR analysis showed that PTPN14 mRNA expression levels were comparable between TA and AA (Figure S1A). En face immunofluorescence staining further revealed significantly higher endothelial PTPN14 levels in the outer curvature of the AA and in the TA compared with the inner curvature of the AA (Figure 1D,E). To assess PTPN14 expression during atherogenesis, *ApoE^−/−^* mice were fed a Western diet for 8 weeks. Consistently, PTPN14 expression was significantly reduced in atherosclerotic intimal regions compared with regions from mice maintained on a normal diet (Figure 1F,G). We further evaluated PTPN14 expression in human coronary arteries and found that endothelial PTPN14 levels were markedly lower in atherosclerotic plaques than in non‐plaque regions (Figure 1H,I).

**FIGURE 1:**
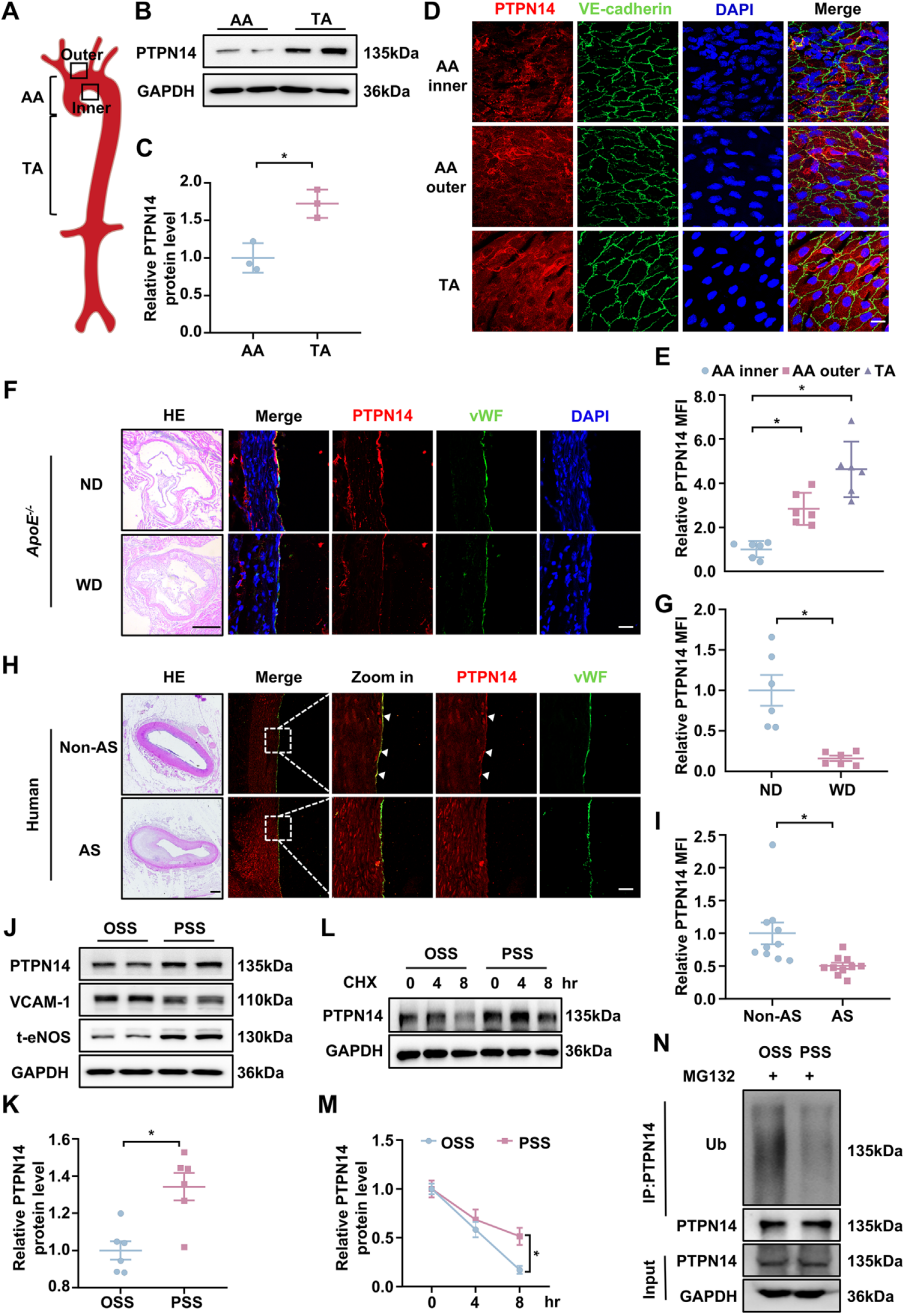
PTPN14 protein levels and stability are regulated by distinct flow patterns. (A‐C) Representative immunoblots (B) and densitometric quantification (C) of PTPN14 expression in intima from the thoracic aorta (TA) and aortic arch (AA) of *ApoE^−/−^* mice. Protein levels were normalized to GAPDH and expressed relative to the AA group. Aortic intima from 2 mice were pooled as 1 sample. Data are mean ± SEM, ^*^ *p *< 0.05 (2‐tailed unpaired Student's *t*‐test), *n* = 3 pooled samples. (D,E) Representative en face immunofluorescence images (D) of PTPN14, VE‐cadherin, and DAPI, and quantification (E) of PTPN14 expression in the inner curvature of the AA (AA inner), outer curvature of the AA (AA outer), and TA of *ApoE^−/−^* mice. Scale bar, 10 µm. PTPN14 fluorescence intensity was normalized to DAPI and expressed relative to AA inner. Data are mean ± SEM, ^*^ *p *< 0.05 (2‐tailed unpaired Student's *t* test), *n* = 6 mice per group. (F‐G) Representative hematoxylin and eosin (HE) staining and immunofluorescence images (F) of aortic rings from *ApoE^−/−^* mice fed a normal diet (ND) or Western diet (WD) for 8 weeks, and quantification (G) of PTPN14 expression within the vWF‐positive area. Scale bars, 500 µm (HE) and 20 µm (immunofluorescence). Data are mean ± SEM, ^*^ *p *< 0.05 (2‐tailed unpaired Student's *t* test), *n* = 6 mice. (H,I) Representative HE and immunofluorescence images (H) of human coronary arteries with or without atherosclerotic plaque, and quantification (I) of PTPN14 expression within the vWF‐positive area. Scale bars, 500 µm (HE) and 20 µm (immunofluorescence). Data are mean ± SEM, ^*^ *p *< 0.05 (2‐tailed Mann‐Whitney *U* test), *n* = 10. (J,K) HUVECs were exposed to oscillatory shear stress (OSS; 0.5 ± 4 dyn/cm^2^, 1 Hz) or pulsatile shear stress (PSS; 12 ± 4 dyn/cm^2^, 1 Hz) for 12 h. Representative immunoblots of PTPN14, VCAM‐1 and total eNOS (t‐eNOS) (J), and quantification of PTPN14 protein levels (K). Data are mean ± SEM, ^*^ *p *< 0.05 (2‐tailed unpaired Student's *t* t‐est), *n* = 6 independent experiments. (L‐M) HUVECs were exposed to OSS or PSS for 8 h and treated with cycloheximide (CHX; 50 µg/mL) for the indicated times, followed by immunoblot analysis of PTPN14 protein levels. Data are mean ± SEM, ^*^ *p *< 0.05 (two‐way ANOVA with Tukey's multiple comparison posthoc test), *n* = 5 independent experiments. (N) HUVECs were exposed to OSS or PSS and meanwhile treated with MG132 (10 µM) for 6 h. PTPN14 was immunoprecipitated, and ubiquitination was assessed by immunoblotting. Data are mean ± SEM; *n* = 3 independent experiments.

To determine whether endothelial PTPN14 responds directly to distinct flow patterns, we next established an in vitro flow system. Consistent to the in vivo data, PTPN14 mRNA expression levels were not significantly altered under pulsatile shear stress (PSS; 12 ± 4 dyn/cm^2^, 1 Hz) or oscillatory shear stress (OSS; 0.5 ± 4 dyn/cm^2^, 1 Hz) (Figure S1B). In contrast, PTPN14 protein levels were significantly reduced under OSS compared with PSS (Figure 1J,K), suggesting a post‐transcriptional regulation. To examine whether PTPN14 protein stability differed under distinct flow conditions, HUVECs were exposed to OSS or PSS and subsequently treated with cycloheximide (CHX, 50 µg/mL) for the indicated time intervals. Compared with PSS, OSS exposure markedly reduced PTPN14 protein stability (Figure 1L,M). We and others have previously reported that PTPN14 undergoes ubiquitination‐proteasome degradation pathway [14, 16]. Consistently, treatment with the proteasome inhibitor MG132, but not the lysosomal inhibitor chloroquine, blocked the shear flow pattern‐dependent regulation of PTPN14 protein levels (Figure S1C,D). Together, these in vivo and in vitro data demonstrate that endothelial PTPN14 protein abundance and stability are dynamically regulated in accordance with hemodynamic shear stress patterns.

### 2.2 Endothelial‐Specific PTPN14 Deficiency Exacerbates Endothelial Inflammation and Atherosclerosis

Global PTPN14 knockout in mice has been reported to compromise viability and to be associated with lymphatic edema. Besides, PTPN14 variants cause defects in organ and tissue development in zebrafish embryos [17, 18]. To investigate the role of PTPN14 in endothelial activation and atherogenesis in vivo, we generated tamoxifen‐inducible, EC‐specific PTPN14 knockout mice (*Ptpn14^iECKO^; Ptpn14^flox/flox^Cdh5‐Cre^+^*) and littermate control mice (*Ptpn14^fl/fl^; Ptpn14^flox/flox^Cdh5‐Cre^−^*). Successful recombination and endothelial‐specific deletion of PTPN14 were confirmed by genotyping PCR (Figure S2A) and en face immunofluorescence staining of the aorta (Figure S2B,C). PTPN14 protein levels remained unaltered in nonvascular tissues including the lung, kidney, and liver between the *Ptpn14^iECKO^* mice and controls (Figure S2D,E). No overt phenotypic abnormalities were observed in *Ptpn14^iECKO^* mice. Next, we crossed *Ptpn14^iECKO^* and *Ptpn14^fl/fl^* mice with *ApoE* ^−/−^ mice to generate double‐knockout (*Ptpn14^iECKO^ApoE^−/−^*, *Ptpn14^fl/fl^ApoE^−/−^*) mice. Eight‐week‐old *Ptpn14^iECKO^ApoE^−/−^* and *Ptpn14^fl/fl^ApoE^−/−^* mice were fed a high‐fat diet for 12 weeks to induce atherosclerosis. En face Oil Red O staining revealed that EC‐specific deletion of PTPN14 significantly increased atherosclerotic plaque burden throughout the aorta compared with control mice (Figure 2A,B). Consistently, PTPN14 deficiency markedly increased lesion area and lipid deposition in the aortic root, without significantly affecting collagen content (Figure 2C,D). Immunohistochemical analysis further showed that endothelial PTPN14 deletion significantly increased macrophage accumulation in aortic root lesions, while the area of α‐smooth muscle actin (α‐SMA)‐positive vascular smooth muscle cells (VSMCs) was not significantly altered (Figure 2E,F). To evaluate whether endothelial PTPN14 deficiency influences systemic metabolic risk factors, we assessed body weight and plasma lipid profiles. No significant differences were observed between the two groups (Figure S2F,G), suggesting that EC‐specific PTPN14 deletion does not alter systemic confounding factors of atherosclerosis.

**FIGURE 2:**
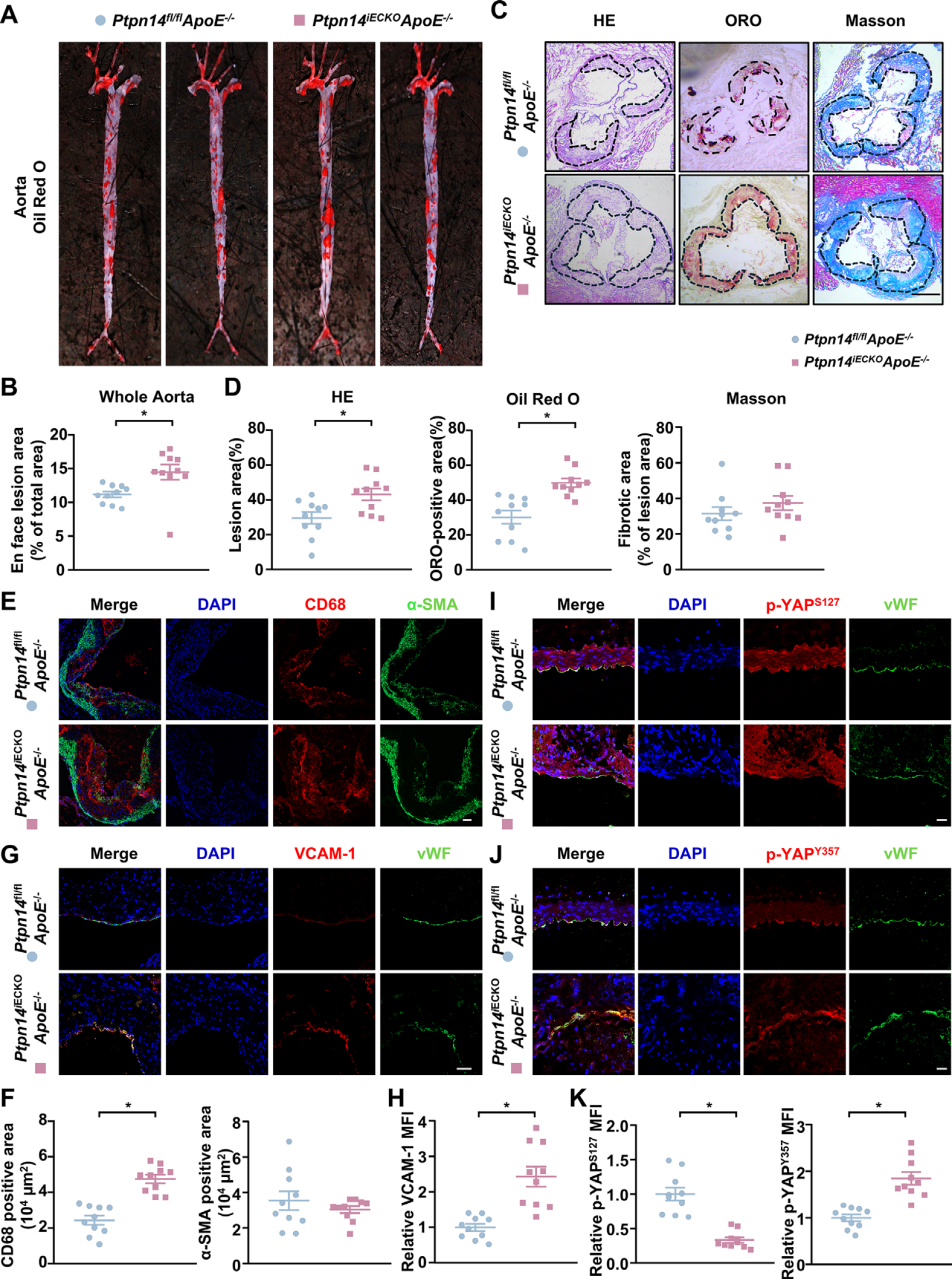
EC‐specific PTPN14 deficiency aggravates endothelial inflammatory response and atherosclerosis. Eight‐week‐old male *Ptpn14^fl/fl^ApoE^−/−^* and *Ptpn14^iECKO^ApoE^−/−^* mice were fed a high‐fat diet for 12 weeks. Arterial tissues and aortic roots were isolated to examine atherosclerotic lesions. (A) Representative en face Oil Red O staining of the whole aorta from the indicated groups. (B) Quantification of aortic lesion area (Oil Red O‐positive) in the whole aorta. Data are mean ± SEM, ^*^ *p *< 0.05 (2‐tailed Mann‐Whitney *U* test), *n* = 10 mice per group. (C) Representative images of hematoxylin and eosin (HE), Oil Red O, and Masson's trichrome staining of aortic root sections. Scale bar, 500 µm. (D) Quantification of lesion size, Oil Red O‐positive area, and collagen content (Masson's trichrome) in aortic root sections. Data are mean ± SEM, ^*^ *p *< 0.05 (2‐tailed unpaired Student's *t* test), *n* = 10 mice per group. (E–K) Representative immunofluorescence images of CD68, α‐smooth muscle actin (α‐SMA), von Willebrand factor (vWF), vascular cell adhesion molecule‐1 (VCAM‐1), p‐YAP^S127^, p‐YAP^Y357^, and DAPI in aortic root sections. Scale bar, 50 µm (CD68 and α‐SMA), 20 µm (vWF, VCAM‐1, p‐YAP^S127^ and p‐YAP^Y357^). Quantification of the CD68‐positive and α‐SMA‐positive areas. VCAM‐1/p‐YAP fluorescence intensity was normalized to DAPI and the relative expression values were compared to that of the *Ptpn14^fl/fl^ApoE^−/−^* group. Data are mean ± SEM, ^*^ *p *< 0.05 (2‐tailed unpaired Student's *t* test), *n* = 10 mice per group.

Given the critical role of endothelial adhesion molecules in leukocyte recruitment, we next assessed vascular cell adhesion molecule 1 (VCAM‐1) expression in the endothelium of aortic roots. Endothelial PTPN14 deficiency resulted in a significant increase in VCAM‐1 expression within the aortic endothelium (Figure 2G,H). Consistent with this pro‐inflammatory phenotypic shift, loss of endothelial PTPN14 led to marked activation of YAP in ECs, as evidenced by reduced phosphorylation of YAP at Ser127 and increased phosphorylation at Tyr357 (Figure 2I,K). Together, these results demonstrate that EC‐specific PTPN14 deficiency enhances endothelial inflammatory activation and exacerbates atherosclerotic lesion development in mice.

### 2.3 E3 Ubiquitin Ligase TRIM21 is a Potential Binding Partner of PTPN14

E3 ubiquitin ligases are key regulators of protein homeostasis by shaping the ubiquitination status of target proteins and thereby controlling their stability and turnover [19]. To identify factors that regulate PTPN14 protein stability, we performed immunopurification followed by mass spectrometry‐based proteomic analysis to screen for proteins associated with PTPN14 in ECs (Figure 3A). Proteomic analysis identified TRIM21 as a prominent PTPN14‐interacting protein, with 15 unique peptides detected and a sequence coverage of 32.42%, suggesting that the E3 ubiquitin ligase TRIM21 is a potential binding partner of PTPN14 (Figure S3A). To validate this interaction, Flag‐tagged PTPN14 and Myc‐tagged TRIM21 were co‐expressed in HEK293T cells, and co‐immunoprecipitation assays confirmed their association (Figure 3B). Endogenous co‐immunoprecipitation further demonstrated an interaction between TRIM21 and PTPN14 in ECs (Figure 3C,D). Moreover, immunofluorescence staining revealed substantial colocalization of TRIM21 and PTPN14 in ECs (Figure 3E,F).

**FIGURE 3:**
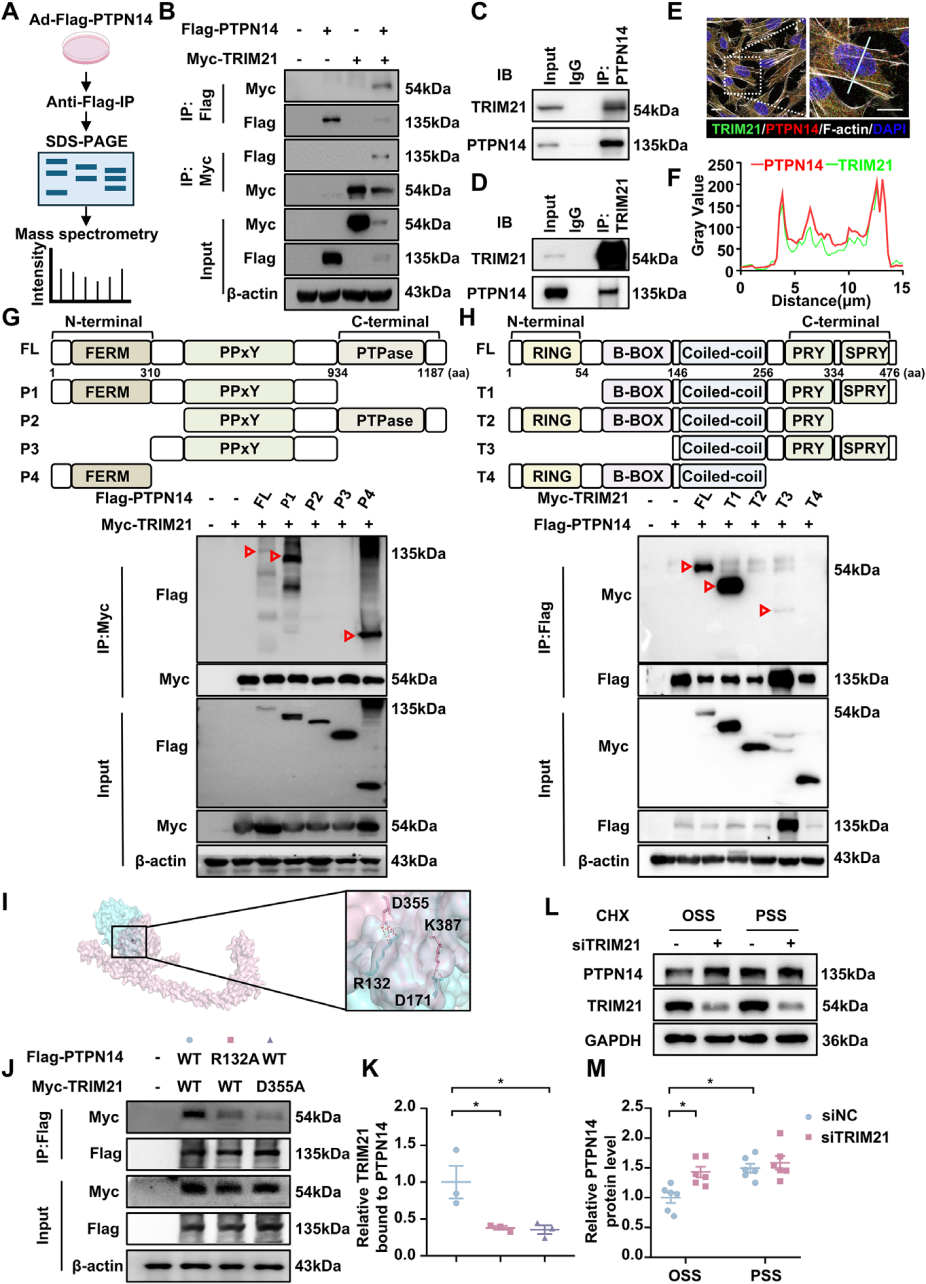
TRIM21 binds to PTPN14 and mediates shear stress‐induced PTPN14 degradation in EC. (A) HUVECs were infected with adenovirus expressing Flag‐tagged PTPN14 (Ad‐Flag‐PTPN14) for 48 h. Protein complexes were affinity‐purified from whole‐cell lysates using anti‐Flag beads and analyzed by mass spectrometry to identify PTPN14‐interacting proteins. (B) Co‐immunoprecipitation (co‐IP) confirming the interaction between Flag‐PTPN14 and Myc‐tagged TRIM21 (Myc‐TRIM21) in cotransfected HEK293T cells. *n* = 3 independent experiments. (C‐D) Endogenous co‐immunoprecipitation in HUVECs was performed using an anti‐PTPN14 or anti‐TRIM21 antibody, and the presence of TRIM21 and PTPN14 in the immunoprecipitants was detected by immunoblotting. Representative blots are shown. *n* = 3 independent experiments. (E) Representative immunofluorescence images showing colocalization of PTPN14 and TRIM21 in HUVECs. Scale bar, 20 µm. *n* = 6 independent experiments. (F) Comparison of the fluorescence intensity of PTPN14 and TRIM21 (white line on the enlarged image) of E. (G) Schematic of PTPN14 truncation constructs. Co‐IP assays in HEK293T cells cotransfected with Myc‐TRIM21 and Flag‐tagged PTPN14 fragments were performed to map the TRIM21‐binding region of PTPN14. *n* = 3 independent experiments. Red triangles indicate target bands. (H) Schematic of TRIM21 truncation constructs. Co‐IP assays in HEK293T cells cotransfected with Flag‐PTPN14 and Myc‐tagged TRIM21 fragments were performed to map the PTPN14‐binding region of TRIM21. *n* = 3 independent experiments. Red triangles indicate target bands. (I) Predicted PTPN14‐TRIM21 interface generated using AlphaFold3 and visualized with PyMOL. The FERM domain of PTPN14 and full‐length of TRIM21 residues are shown as blue and pink sticks, respectively. R132 of PTPN14 is predicted to form a salt bridge with D355 of TRIM21. (J) HEK293T cells were transfected with Flag‐PTPN14‐WT or Flag‐PTPN14‐R132A together with Myc‐TRIM21‐WT or Myc‐TRIM21‐D355A. PTPN14‐associated TRIM21 was assessed by immunoprecipitation with anti‐Flag beads followed by immunoblotting. (K) Quantitative analysis of TRIM21‐PTPN14 interaction by immunoprecipitation. Data are mean ± SEM, ^*^ *p *< 0.05 (2‐tailed unpaired Student's *t*‐test), *n* = 3 independent experiments. (L‐M) HUVECs were transfected with negative control siRNA (siNC) or TRIM21 siRNA (siTRIM21) for 36 h, then exposed to oscillatory shear stress (OSS) or pulsatile shear stress (PSS) in the presence of cycloheximide (CHX; 50 µg/mL) for 6 h. Representative immunoblots of PTPN14 are shown. Data are mean ± SEM, ^*^ *p *< 0.05 (two‐way ANOVA with Tukey's multiple comparison post‐hoc test), *n* = 6 independent experiments.

TRIM21, similar to other TRIM family members, contains an N‐terminal RING finger domain, a B‐box domain, a coiled‐coil domain, and a C‐terminal PRY/SPRY domain [20]. PTPN14 is composed of an N‐terminal FERM domain, a central region, and a C‐terminal protein tyrosine phosphatase (PTP) domain [9]. To map the interaction domains, we co‐expressed a series of Flag‐tagged PTPN14 truncation mutants together with full‐length Myc‐tagged TRIM21 in HEK293T cells. Anti‐Myc co‐immunoprecipitation showed that the FERM domain of PTPN14 mediated binding to TRIM21, whereas the PPxY‐containing region and the PTP domain did not (Figure 3G). Conversely, domain‐mapping experiments using Myc‐tagged TRIM21 truncation mutants revealed that the SPRY domain of TRIM21 was responsible for binding to full‐length PTPN14, while the RING, B‐box, and coiled‐coil domains were dispensable for this interaction (Figure 3H). To gain structural insight into the interaction between PTPN14 and TRIM21, we employed AlphaFold3 to predict the binding interface. The model suggested that Arg132 (R132) in the PTPN14 FERM domain forms a critical interaction with Asp355 (D355) in the TRIM21 SPRY domain, likely via a salt bridge (Figure 3I). Consistent with this prediction, introducing an alanine substitution at either PTPN14 R132 (R132A) or TRIM21 D355 (D355A) disrupted the stable association between the two proteins (Figure 3J,K). Together, these results indicate that R132 in the PTPN14 FERM domain and D355 in the TRIM21 SPRY domain constitute the key interface mediating their interaction. Functionally, TRIM21 silencing significantly increased PTPN14 protein levels under OSS and abolished the differential regulation of PTPN14 protein abundance between distinct flow patterns (Figure 3L,M), which effects were further validated using an alternative siRNA sequence against TRIM21 (Figure S3B,C). Collectively, these results demonstrate that TRIM21 binds to PTPN14 and serves as the key E3 ubiquitin ligase mediating flow‐dependent ubiquitination and degradation of PTPN14 in ECs.

### 2.4 TRIM21 Mediates K48‐Linked Ubiquitination of PTPN14 at Lysine 956

We next investigated whether TRIM21 regulates PTPN14 protein stability. Overexpression of TRIM21 significantly reduced the stability of the PTPN14 protein (Figure 4A). Consistently, TRIM21 overexpression decreased PTPN14 protein levels in a dose‐dependent manner, and this effect was abolished by treatment with the proteasome inhibitor MG132, indicating that TRIM21 regulates PTPN14 through a proteasome‐dependent pathway (Figure 4B).

**FIGURE 4:**
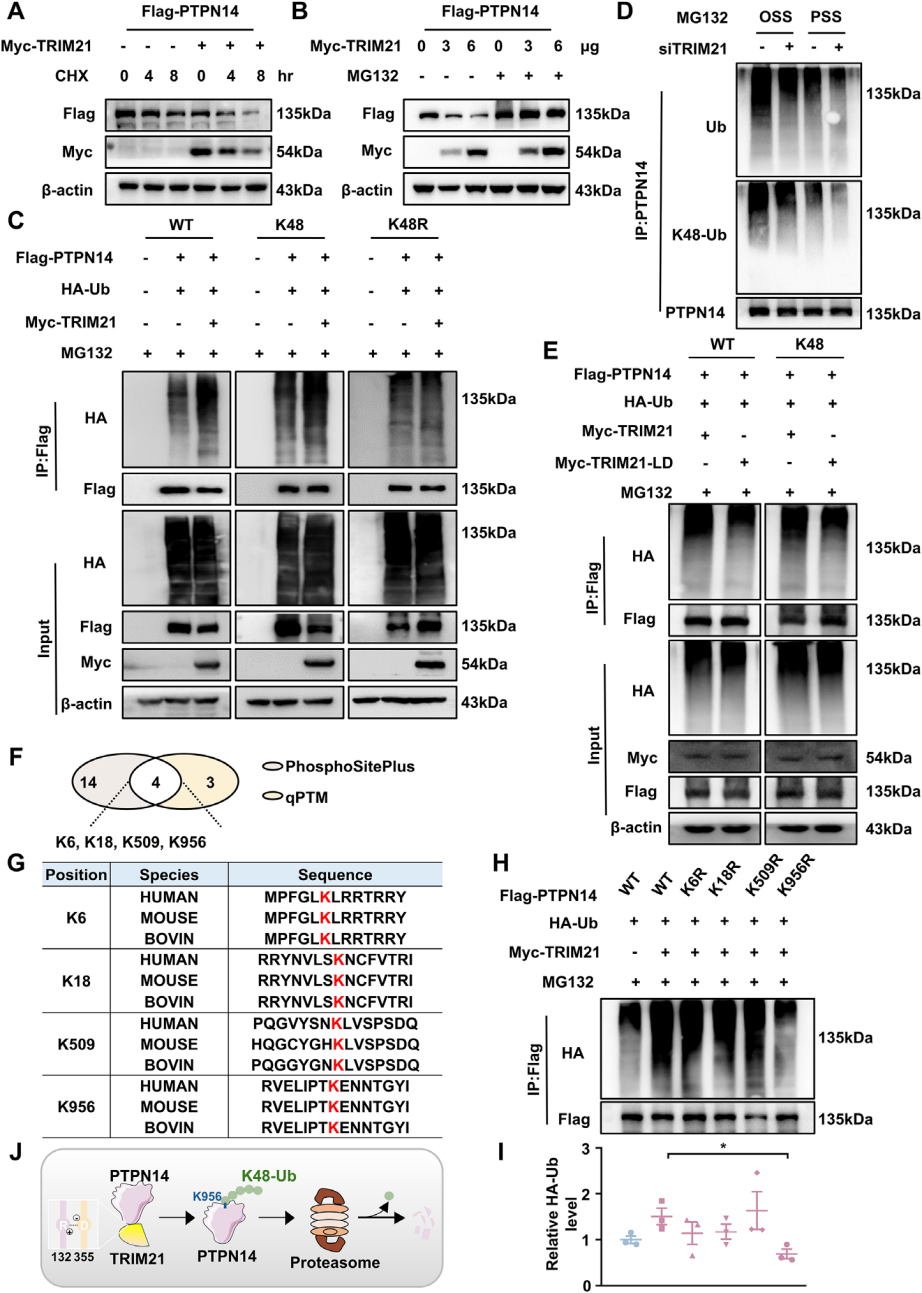
TRIM21 mediates PTPN14 ubiquitination and degradation. (A) HEK293T cells expressing Flag‐tagged PTPN14 (Flag‐PTPN14) with or without Myc‐tagged TRIM21 (Myc‐TRIM21) were treated with cycloheximide (CHX; 50 µg/mL) for the indicated times. Whole‐cell lysates were analyzed by immunoblotting. *n* = 6 independent experiments. (B) HEK293T cells were transfected with Flag‐PTPN14 and increasing amounts of Myc‐TRIM21 (0, 3, and 6 µg) and treated with vehicle or the proteasome inhibitor MG132 (10 µM). Flag‐PTPN14 protein levels were assessed by immunoblotting. *n* = 6 independent experiments. (C) HEK293T cells were cotransfected with Flag‐PTPN14, Myc‐TRIM21, and HA‐tagged ubiquitin (HA‐Ub; WT, K48‐only, or K48R) and treated with MG132 (10 µM). Flag‐PTPN14 was immunoprecipitated (IP: Flag) and immunoblotted with indicated antibodies. *n* = 3 independent experiments. (D) HUVECs were transfected with negative control siRNA (siNC) or TRIM21 siRNA (siTRIM21) for 36 h, then exposed to OSS or PSS in the presence of MG132 (10 µM) for 6 h. PTPN14 was then immunoprecipitated and analyzed by immunoblotting to assess total polyubiquitination and K48‐linked ubiquitination. Representative blots are shown. *n* = 3 independent experiments. (E) HEK293T cells expressing Flag‐PTPN14 and HA‐Ub (WT or K48‐only) were cotransfected with Myc‐TRIM21 or an E3 ligase‐dead mutant (Myc‐TRIM21‐LD) and treated with MG132 (10 µM). Flag‐PTPN14 was immunoprecipitated (IP: Flag) and immunoblotted with indicated antibodies. *n* = 3 independent experiments. (F) Venn diagram summarizing predicted PTPN14 ubiquitination sites compiled from PhosphoSitePlus and the qPTM database. (G) Amino acid sequence alignment of PTPN14 orthologs between human, mouse, and bovine species. (H,I) Ubiquitination assays of Flag‐PTPN14 WT and the indicated lysine‐to‐arginine mutants (K6R, K18R, K509R, and K956R) were performed in the presence of HA‐Ub with MG132 (10 µM) treatment. Flag‐PTPN14 was immunoprecipitated (IP: Flag) and ubiquitination levels were detected by anti‐HA immunoblotting. Data are mean ± SEM, ^*^ *p *< 0.05 (one‐way ANOVA with Tukey's multiple comparison posthoc test), *n* = 3 independent experiments. (J) Schematic illustration of the proposed mechanism. TRIM21 interacts with PTPN14 via residues R132 and D355 and acts as an E3 ubiquitin ligase to catalyze K48‐linked polyubiquitination of PTPN14 at lysine 956, thereby promoting its proteasomal degradation.

To directly assess whether TRIM21 mediates PTPN14 ubiquitination, HEK293T cells were co‐transfected with Flag‐tagged PTPN14, Myc‐tagged TRIM21, and HA‐tagged ubiquitin, followed by MG132 treatment. Flag‐PTPN14 was immunoprecipitated and immunoblotted with anti‐HA antibody to detect ubiquitination. TRIM21 markedly increased the polyubiquitination of PTPN14 (Figure 4C). Because K48‐ and K63‐linked ubiquitin chains represent the two most abundant linkage types in cells and play distinct roles in proteasomal degradation and signal transduction [21], we next determined the ubiquitin linkage specificity of TRIM21‐mediated PTPN14 ubiquitination. HEK293T cells were co‐transfected with Flag‐PTPN14, Myc‐TRIM21, and HA‐tagged ubiquitin mutants (K48‐only, K48R, K63‐only, and K63R) and treated with MG132. TRIM21 robustly promoted K48‐linked, but not K63‐linked, ubiquitination of PTPN14, as evidenced by increased ubiquitination in the presence of K48‐only and K63R ubiquitin, but not K63‐only or K48R ubiquitin (Figure 4C and Figure S4A). Consistently, in the presence of the proteasome inhibitor MG132, TRIM21 knockdown markedly reduced the total and K48‐linked ubiquitination levels of PTPN14 under OSS conditions, but not under PSS (Figure 4D), indicating that TRIM21 mediates OSS‐induced K48‐linked ubiquitination and degradation of PTPN14.

To determine whether TRIM21‐mediated ubiquitination of PTPN14 depends on its E3 ligase activity, we generated a ligase‐dead TRIM21 mutant (C16A, C31A, and H33W), as previously reported [22, 23]. Co‐immunoprecipitation assays revealed that both total ubiquitination and K48‐linked ubiquitination of PTPN14 induced by TRIM21 were abolished by the ligase‐dead mutation, indicating that the E3 ligase activity of TRIM21 is indispensable for PTPN14 ubiquitination (Figure 4E).

To identify the ubiquitination site(s) on PTPN14 targeted by TRIM21, we used PhosphoSitePlus [24] and qPTM [25, 26] databases to predict potential ubiquitinated lysine residues (Tables S3 and S4). Intersection analysis identified four candidate lysine sites (K6, K18, K509, and K956) (Figure 4F), all of which are highly conserved among human, mouse, and bovine PTPN14 sequences (Figure 4G). We next generated lysine‐to‐arginine mutants at each of these sites and expressed them in HEK293T cells. Notably, mutation of K956 (K956R), but not mutations at K6, K18, or K509, markedly attenuated TRIM21‐induced ubiquitination of PTPN14 (Figure 4H,I), indicating that lysine 956 is the major ubiquitination site targeted by TRIM21. Together, these data demonstrate that TRIM21 mediates proteasome‐dependent degradation of PTPN14 by promoting K48‐linked ubiquitination at lysine 956 (Figure 4J).

### 2.5 TRIM21 Undergoes Flow Pattern‐Dependent Phase Separation to Form Co‐Condensates With PTPN14

Next, we sought to elucidate the mechanism underlying TRIM21‐mediated PTPN14 degradation under distinct flow patterns. Notably, TRIM21 protein levels were not significantly altered by different flow conditions (Figure S5A,B). Unexpectedly, immunofluorescence staining revealed a prominent punctate cytoplasmic distribution of TRIM21 in ECs (Figure 5A). While prolonged exposure to PSS did not affect the number of TRIM21 condensates, OSS induced a time‐dependent increase in TRIM21 puncta as early as 6 h after stimulation (Figure 5A,B). Consistently, en face immunofluorescence staining of the aorta from ApoE^−/−^ mice indicated that TRIM21 condensates were markedly enriched in the atheroprone AA region compared to the atheroprotected TA region (Figure S5C,D). Given emerging evidence that LLPS in ECs is tightly coupled to shear stress signaling [27], these distinct biophysical patterns led us to hypothesize that TRIM21 undergoes shear stress‐responsive liquid‐liquid phase separation (LLPS). The coiled‐coil (CC) domain of tripartite motif (TRIM) proteins is essential for condensate formation, and disease‐associated single‐nucleotide polymorphisms within this domain impair phase separation [28]. Consistent with this notion, OSS‐induced TRIM21 condensate formation was abolished upon deletion of the CC domain (Figure S5E,F), indicating that TRIM21 undergoes LLPS in a CC domain‐dependent manner in ECs. To directly assess whether TRIM21 exhibits LLPS behavior in ECs, we ectopically expressed mCherry‐tagged TRIM21 in ECs. Time‐lapse live‐cell imaging demonstrated that mCherry‐TRIM21 puncta underwent dynamic fusion and fission events, a hallmark of liquid‐like condensates (Figure 5C). Furthermore, treatment with 1,6‐hexanediol (1,6‐HD), a disruptor of liquid‐like assemblies, markedly reduced and dissolved TRIM21 puncta, whereas 2,5‐hexanediol (2,5‐HD), used as a negative control, had no effect (Figure 5D,E).

**FIGURE 5:**
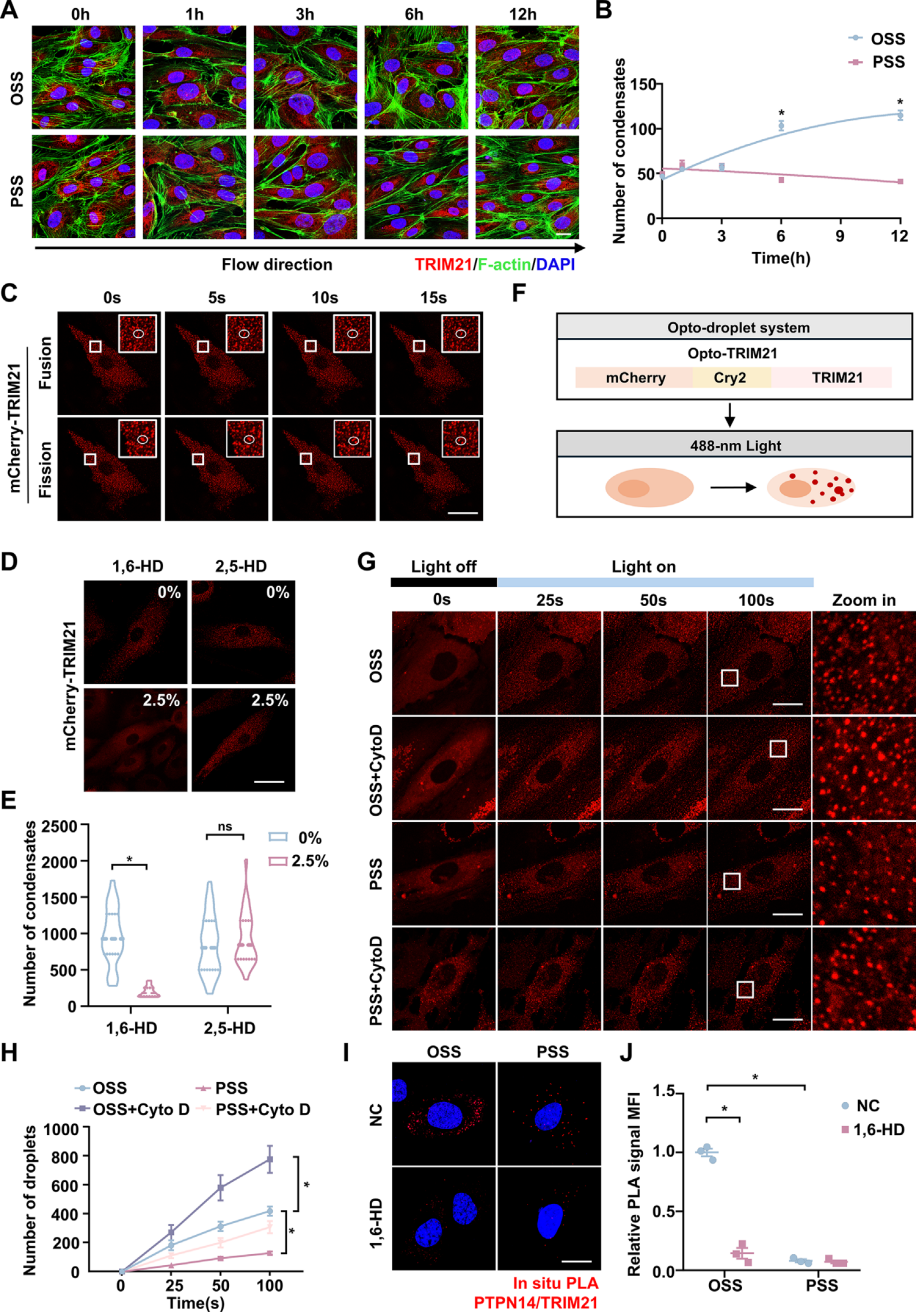
TRIM21 undergoes flow pattern‐dependent phase separation to form co‐condensates with PTPN14. (A) Representative immunofluorescence of TRIM21 in HUVECs subjected to OSS (0.5 ± 4 dyn/cm^2^, 1 Hz) or PSS (12 ± 4 dyn/cm^2^, 1 Hz) for indicated time periods (0, 1, 3, 6, and 12 h), Scale bar, 10 µm. (B) Quantification of TRIM21 condensates. Data are mean ± SEM, ^*^ *p *< 0.05 (two‐way ANOVA with Tukey's multiple comparison post‐hoc test), *n* = 6 independent experiments. (C) Time‐lapse images of HUVECs expressing mCherry‐TRIM21; puncta fusion and fission are shown (boxed). Scale bar, 20 µm. (D,E) Representative images and quantification of mCherry‐TRIM21 condensates in HUVECs treated with 2.5% 1,6‐hexanediol (1,6‐HD) or 2,5‐hexanediol (2,5‐HD). Scale bar, 20 µm. Data are mean ± SEM, ^*^ *p *< 0.05 (2‐tailed unpaired Student's *t*‐test), *n* = 6 independent experiments. (F) Schematic illustration of the OptoDroplet assay. Candidate gene (TRIM21) was fused with mCherry and Cry2. Upon blue light exposure, proteins with phase‐separation capacity formed condensates in cells. (G,H) OptoDroplet assay and quantification of Opto‐TRIM21 in HUVECs exposed to OSS, PSS, or Cyto D treatment followed by OSS and PSS exposure, Scale bar, 20 µm. Data are mean ± SEM, ^*^ *p *< 0.05 (two‐way ANOVA with Tukey's multiple comparison posthoc test), *n* = 6 independent experiments. (I,J) Proximity ligation assay (PLA) analysis showing TRIM21‐PTPN14 association in OSS, PSS, and treated with 2.5% 1,6‐hexanediol (1,6‐HD). Nuclei were stained with DAPI (blue), and TRIM21‐PTPN14 interactions were visualized with in situ PLA signals (red). Scale bar, 20 µm. Data are mean ± SEM, ^*^ *p *< 0.05 (two‐way ANOVA with Tukey's multiple comparison post‐hoc test), *n* = 3 independent experiments.

To further interrogate the flow‐dependent regulation of TRIM21 phase separation, we employed an OptoDroplet (Opto‐) system, in which TRIM21 was fused to the light‐sensitive oligomerization domain of cryptochrome 2 (Cry2) to enable spatiotemporal control of LLPS (Figure 5F). Notably, compared with PSS, blue‐light stimulation robustly induced Opto‐TRIM21 droplet formation under OSS conditions (Figure 5G,H). Previous studies have shown that stress fiber bundling spatially sequesters TRIM21 on actin filaments, thereby limiting its accessibility to substrate(s) [29]. Given that shear stress rapidly induces cytoskeletal remodeling in ECs [30], we hypothesized that actin reorganization might underlie the differential LLPS behavior of TRIM21 under distinct flow patterns. To test this, we treated cells with cytochalasin D (Cyto D), a potent inhibitor of actin polymerization, to disrupt cytoskeletal integrity. Notably, Cyto D treatment markedly enhanced blue‐light‐induced TRIM21 droplet formation (Figure 5G,H).

Finally, proximity ligation assays (PLA) demonstrated that OSS significantly enhanced the spatial proximity between TRIM21 and PTPN14, an effect that was abolished by 1,6‐HD treatment (Figure 5I,J), indicating TRIM21 LLPS facilitates the interaction between TRIM21 and PTPN14. Collectively, these results indicate that TRIM21 undergoes LLPS in ECs in a shear stress‐dependent manner, driven by cytoskeletal remodeling, and that this phase separation facilitates the recruitment and interaction of PTPN14, thereby promoting its degradation under disturbed flow.

### 2.6 TRIM21 Overexpression Exacerbates Disturbed Flow‐Induced Endothelial Activation and Atherosclerosis

Next, we investigated the role of TRIM21 in endothelial activation. Compared with PSS, exposure to OSS significantly induced the mRNA expression of pro‐inflammatory genes, including VCAM‐1 and ICAM‐1, in ECs. Notably, this OSS‐induced inflammatory response was further aggravated by TRIM21 overexpression (Figure S6A,B). Consistently, OSS exposure markedly enhanced THP‐1 monocyte adhesion to ECs, and this effect was significantly exacerbated by TRIM21 overexpression (Figure S6C,D).

To determine the pro‐atherogenic role of TRIM21 in vivo, we employed a partial carotid artery ligation model in *Ldlr^−/−^* mice. The left carotid arteries were locally infected with Ad‐NC or Ad‐TRIM21, followed by high‐fat diet feeding for 2 weeks after surgery. En face immunofluorescence staining confirmed successful TRIM21 overexpression in the ligated carotid arteries (Figure S6E). HE staining revealed that TRIM21 overexpression significantly increased lesion formation in the ligated carotid arteries (Figure S6F,G). Immunofluorescence analysis further demonstrated that TRIM21 overexpression reduced endothelial PTPN14 levels while concomitantly increasing VCAM‐1 expression in the ligated carotid arteries (Figure S6H–K). Collectively, these findings indicate that TRIM21 overexpression exacerbates disturbed flow‐induced endothelial inflammatory activation and promotes atherosclerosis.

### 2.7 Endothelial‐Specific TRIM21 Deficiency Attenuates Endothelial Inflammation and Atherosclerosis

We next examined the effects of TRIM21 silencing on endothelial activation. In contrast to the pro‐inflammatory effects observed with TRIM21 overexpression, TRIM21 silencing markedly attenuated OSS‐induced mRNA expression of multiple inflammatory genes, including VCAM‐1, ICAM‐1, SELE, and MCP‐1, in cultured ECs (Figure 6A).

**FIGURE 6:**
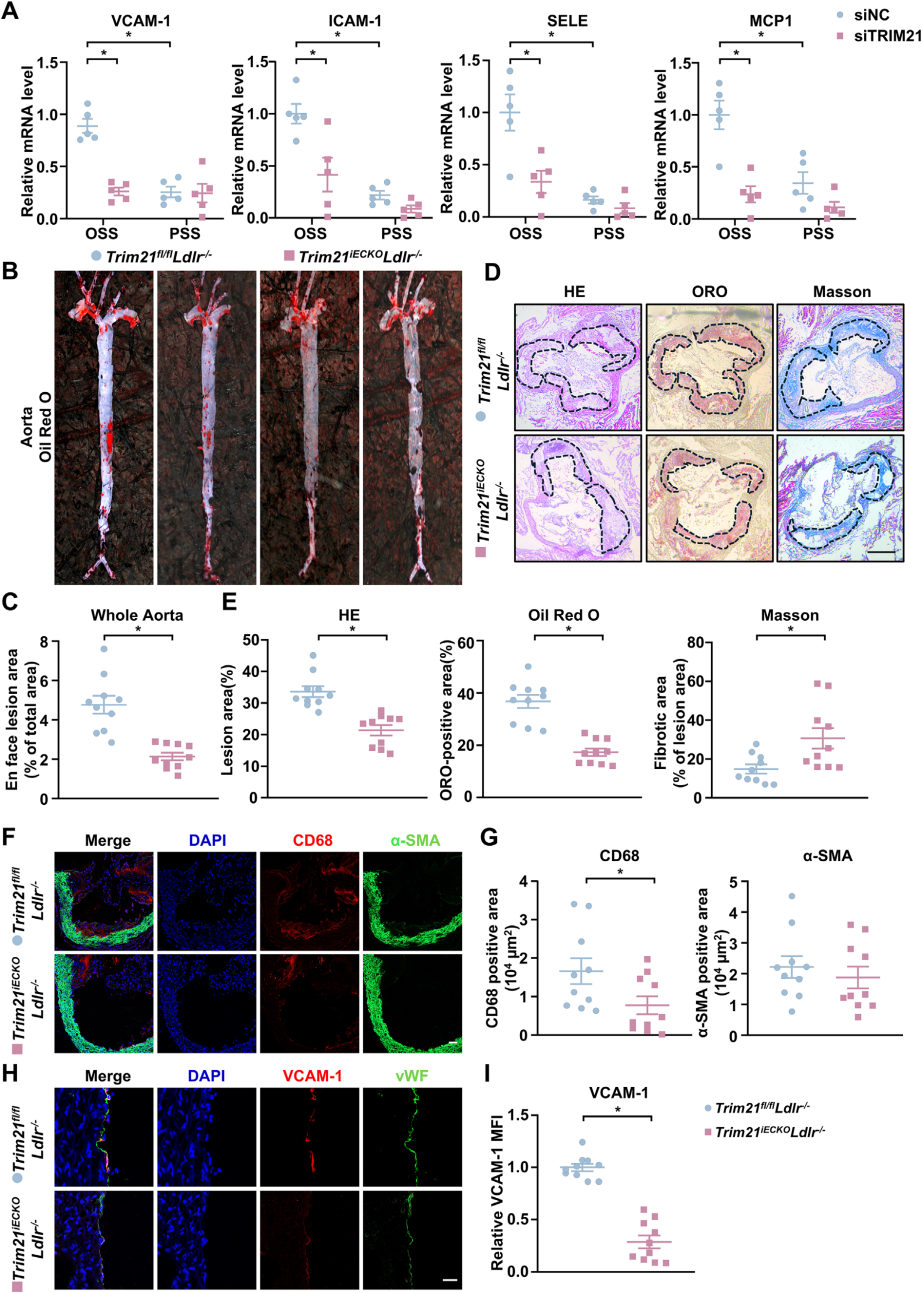
Endothelial TRIM21 deficiency attenuates disturbed flow‐induced atherosclerosis. (A) HUVECs were transfected with negative control siRNA (siNC) or TRIM21 siRNA (siTRIM21) for 36 h, then exposed to OSS (0.5 ± 4 dyn/cm^2^, 1 Hz) or PSS (12 ± 4 dyn/cm^2^, 1 Hz) for 6 h. RT‐qPCR analysis of VCAM‐1, ICAM‐1, SELE, and MCP‐1 mRNA expression is shown with quantification. Data are mean ± SEM, ^*^ *p *< 0.05 (two‐way ANOVA with Tukey's multiple comparison post‐hoc test), *n* = 5 independent experiments. (B‐I) Eight‐week‐old male *Trim21^fl/fl^Ldlr^−/‐ ^* and *Trim21^iECKO^Ldlr^−/−^* mice were fed a high‐fat diet for 12 weeks. Aortas and aortic roots were harvested for assessment of atherosclerotic lesion burden and plaque composition. (B) Representative images of en face Oil‐Red O staining of the aortas. (C) Quantification of Oil Red O‐positive plaque area in the entire aorta. Data are mean ± SEM, ^*^ *p *< 0.05 (2‐tailed unpaired Student's *t* test), *n* = 10 mice per group. (D) Representative staining of aortic root sections by hematoxylin and eosin (HE), Oil‐Red O, and Masson staining of the aortic roots. Scale bars, 500 µm. (E) Quantification of plaque size, Oil‐Red O‐positive area, and collagen fiber content in aortic root sections. Data are mean ± SEM, ^*^ *p *< 0.05 (2‐tailed unpaired Student's *t*‐test), *n* = 10 mice per group. (F‐I) Representative immunofluorescence images of CD68, α‐smooth muscle actin (α‐SMA), von Willebrand factor (vWF), vascular cell adhesion molecule‐1 (VCAM‐1) in aortic root sections, with nuclei counterstained with DAPI. Scale bar, 50 µm (CD68 and α‐SMA), 20 µm (vWF and VCAM‐1). Quantification includes positive areas of CD68 and α‐SMA, and relative VCAM‐1 fluorescence intensity (normalized to DAPI). Data are mean ± SEM, ^*^ *p *< 0.05 (2‐tailed unpaired Student's *t*‐test), *n* = 10 mice per group.

To determine whether endothelial TRIM21 deficiency influences atherogenesis in vivo, we generated tamoxifen‐inducible, EC‐specific TRIM21 knockout mice (*Trim21^iECKO^; Trim21^flox/flox^Cdh5‐Cre^+^*) and their littermate controls (*Trim21^fl/fl^; Trim21^flox/flox^Cdh5‐Cre^−^*) (Figure S7A–C). These mice were subsequently crossed with *Ldlr^−/−^* mice to generate *Trim21^iECKO^Ldlr^−/−^* and *Trim21^fl/fl^Ldlr^−/−^* mice. Notably, because *Trim21* and *ApoE* are both located on mouse chromosome 7, the *Ldlr^−/^* ^−^ background was used to establish the atherosclerosis model. After 12 weeks of high‐fat diet feeding, en face Oil Red O staining revealed that EC‐specific TRIM21 deletion significantly reduced total atherosclerotic lesion area in the aortas of *Trim21^iECKO^Ldlr^−/−^* mice compared with *Trim21^fl/fl^Ldlr^−/−^* controls (Figure 6B,C). Histological analysis of aortic roots further demonstrated that endothelial TRIM21 deficiency significantly reduced lesion size, lipid accumulation, and macrophage infiltration, while having minimal effects on VSMC content (Figure 6D–G). Immunofluorescence analysis further demonstrated that TRIM21 deficiency upregulated endothelial PTPN14 levels while concomitantly decreasing VCAM‐1 expression in the aortic roots (Figure S7D,E and Figure 6H,I). Importantly, body weight and plasma lipid profiles were comparable between the two groups (Figure S7F,G), indicating that the atheroprotective effects of TRIM21 deletion were independent of systemic metabolic changes. Collectively, these findings demonstrate that EC‐specific TRIM21 deficiency alleviates endothelial inflammatory activation and protects against atherosclerosis.

### 2.8 Endothelial PTPN14 Deficiency Blunts the Atheroprotective Effect of TRIM21 Deficiency in Mice

To further establish whether the TRIM21‐PTPN14 regulatory axis plays a critical role in atherosclerosis in vivo, we generated EC‐specific PTPN14 and TRIM21 double‐knockout mice and induced hyperlipidemia by tail‐vein administration of AAV‐PCSK9^D377Y^ [31], followed by 12 weeks of high‐fat diet feeding.

Consistent with the phenotype observed in *Ptpn14^iECKO^* mice, en face Oil Red O staining revealed that endothelial PTPN14 deficiency markedly increased atherosclerotic lesion area throughout the aorta compared with AAV‐PCSK9^D377Y^ treated control mice (Figure 7A,B). Notably, the atheroprotective effect conferred by endothelial TRIM21 deficiency was largely abolished in the absence of PTPN14, as evidenced by comparable lesion areas between *Ptpn14^iECKO^* and *Ptpn14^iECKO^Trim21^iECKO^* mice (Figure 7A,B).

**FIGURE 7:**
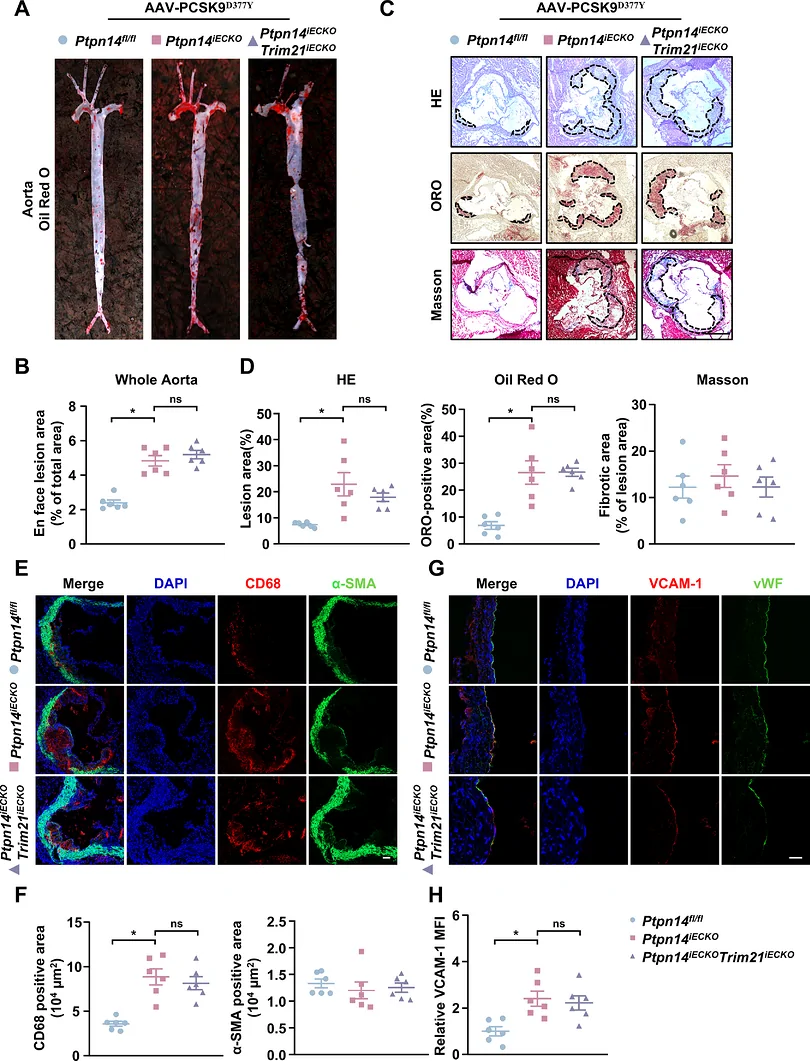
Endothelial PTPN14 deficiency blunts the atheroprotective effect of TRIM21 deficiency in mice. Eight‐week‐old male *Ptpn14^fl/fl^*, *Ptpn14^iECKO^* and *Ptpn14 ^iECKO^Trim21^iECKO ^* mice were fed a high‐fat diet for 12 weeks. Arterial tissues and aortic roots were isolated to examine atherosclerotic lesions. (A) Representative images of en face Oil‐Red O staining of the aortas. (B) Quantification of the plaque area in the whole aorta. Data are mean ± SEM, ^*^ *p *< 0.05 (one‐way ANOVA with Tukey's multiple comparison post‐hoc test), *n* = 6 mice per group. (C) Representative staining of aortic root sections by hematoxylin and eosin (HE), Oil‐Red O, and Masson staining of the aortic roots. Scale bars, 500 µm. (D) Quantification of plaque size, Oil‐Red O‐positive area, and collagen fiber content in aortic root sections. Data are mean ± SEM, ^*^ *p *< 0.05 (one‐way ANOVA with Tukey's multiple comparison post‐hoc test), *n* = 6 mice per group. (E‐H) Representative immunofluorescence images of CD68, α‐smooth muscle actin (α‐SMA), von Willebrand factor (vWF), vascular cell adhesion molecule‐1 (VCAM‐1) in aortic root sections, with nuclei counterstained with DAPI. Scale bar, 50 µm (CD68 and α‐SMA), 20 µm (vWF and VCAM‐1). Quantification includes positive areas of CD68 and α‐SMA, and relative VCAM‐1 fluorescence intensity (normalized to DAPI). Data are mean ± SEM, ^*^ *p *< 0.05 (one‐way ANOVA with Tukey's multiple comparison post‐hoc test), *n* = 6 mice per group.

Histological analysis of aortic roots further confirmed that combined deletion of TRIM21 failed to ameliorate the pro‐atherogenic phenotype caused by PTPN14 deficiency. Compared with *Ptpn14^iECKO^* mice, *Ptpn14^iECKO^Trim21^iECKO^* mice exhibited similar lesion size, lipid accumulation, and collagen deposition (Figure 7C,D). Immunofluorescence staining demonstrated that macrophage infiltration and VSMC content in aortic roots were not significantly altered by TRIM21 deletion under PTPN14‐deficient conditions (Figure 7E,F). Moreover, endothelial expression of the inflammatory adhesion molecule VCAM‐1 was markedly increased in *Ptpn14^iECKO^* mice, and this upregulation was not attenuated by concomitant TRIM21 deletion (Figure 7G,H), indicating that the anti‐inflammatory effect of TRIM21 deficiency is dependent on intact PTPN14 signaling. Importantly, body weight and plasma levels of triglycerides, total cholesterol, and LDL cholesterol were comparable among all experimental groups (Figure S8A,B). Collectively, these in vivo findings demonstrate that the atheroprotective effect of TRIM21 is critically dependent on its regulation of PTPN14, highlighting the TRIM21‐PTPN14 axis as a functionally essential pathway governing endothelial activation and as a promising therapeutic target for atherosclerosis.

## 3 Discussion

Shear stress‐induced endothelial activation represents a critical initiating event in atherogenesis [32], highlighting the importance of identifying endothelial mechanosensors that decode hemodynamic cues. In the present study, we identify endothelial PTPN14 as a previously unrecognized mechanosensitive protein that responds to distinct flow patterns. By generating EC‐specific PTPN14 knockout, TRIM21 knockout, and PTPN14/TRIM21 double‐knockout mouse models and systematically evaluating their atherosclerotic phenotypes, we demonstrate that TRIM21‐mediated ubiquitination and degradation of PTPN14 is a key mechanism driving disturbed flow‐associated endothelial activation and accelerating atherosclerosis progression. Mechanistically, we delineated the interaction mode between TRIM21 and PTPN14 and demonstrated that TRIM21 promotes K48‐linked ubiquitination of PTPN14 at lysine 956, thereby regulating its protein stability. Furthermore, we revealed that TRIM21 undergoes LLPS in ECs, a process markedly enhanced under disturbed flow conditions, which in turn facilitates TRIM21‐dependent PTPN14 degradation. Collectively, these findings uncover a TRIM21‐PTPN14‐dependent mechanotransduction pathway that links pathological shear stress to endothelial dysfunction and atherosclerosis.

PTPN14 is a non‐receptor protein tyrosine phosphatase that is evolutionarily conserved from Drosophila to humans and is best known for its roles in developmental signaling and tumor suppression [9, 17, 33]. Consistent with this function, recurrent mutations in PTPN14 have been identified in multiple human malignancies, including colorectal cancer and basal cell carcinoma, supporting its classification as a putative tumor suppressor [11, 12, 34, 35]. Despite its well‐established roles in development and tumorigenesis, the cardiovascular functions of PTPN14 have remained largely unexplored. We observed that PTPN14 is most abundantly expressed in ECs compared with other vascular wall cell types, suggesting a previously unappreciated role in endothelial biology. In the present study, we identify PTPN14 as a novel mechanosensitive protein that responds to distinct shear stress patterns, and demonstrate that EC‐specific deletion of PTPN14 exacerbates endothelial inflammatory activation and accelerates atherosclerotic progression. Previous studies have shown that inherited variations in human PTPN14 are associated with developmental abnormalities, including dysregulated angiogenesis, impaired lymphatic development, and choanal atresia [17, 33], implying a potential role for PTPN14 in cardiovascular development. In line with this notion, a recent report demonstrated that only approximately 60% of global *Ptpn14* knockout mice survived postnatally, and the surviving mice exhibited a significantly increased heart‐to‐body weight ratio, indicative of abnormal cardiovascular development [36]. Moreover, we recently generated VSMC‐specific PTPN14 knockout mice and similarly observed no apparent defects in vascular development [37]. Under a high‐fat diet challenge, EC‐specific PTPN14 deficiency resulted in heightened endothelial inflammation and markedly accelerated atherosclerosis. These findings suggest that PTPN14 might play a vital role in both vascular development and pathological remodeling. Plaque composition analyses at the aortic root of EC‐PTPN14‐deficient mice further revealed comparable VSMC content but increased macrophage accumulation, suggestive of altered plaque remodeling and potentially reduced plaque stability. Although endothelial inflammation is known to orchestrate intercellular communication among ECs, VSMCs, and immune cells [38], the mechanisms by which endothelial PTPN14 influences other cells within atherosclerotic lesions remain to be elucidated. In addition, our recent observations indicate that PTPN14 overexpression in VSMCs enhances PDGF signaling through PDGFRβ, thereby accelerating injury‐induced VSMC phenotypic switching [37]. Taken together, these findings highlight the context‐dependent and cell type‐specific functions of PTPN14 in the vasculature. Although the present study identifies endothelial PTPN14 as an atheroprotective factor that restrains inflammatory activation, how to achieve spatially and temporally precise targeting of endothelial PTPN14 for the prevention or treatment of atherosclerosis remains an important challenge for future investigation.

High‐risk oncogenic human papillomavirus (HPV) E7 proteins have been reported to interact with the PTP domain of PTPN14 via the C‐terminal region of HPV E7, thereby targeting PTPN14 for proteasome‐mediated degradation [39]. Despite this observation, whether PTPN14 undergoes ubiquitination, as well as the specific ubiquitin linkage types and modification sites involved, has remained unclear. We previously reported that Harmine suppresses the overall ubiquitination level of PTPN14 in ECs [14]. In the present study, we extend these findings by characterizing the ubiquitination pattern of PTPN14 and demonstrate that TRIM21 predominantly mediates K48‐linked, but not K63‐linked, ubiquitination of PTPN14. Through a combination of bioinformatic prediction and mutational analyses of four highly conserved lysine residues (K6, K18, K509, and K956), we identify lysine 956, located within the C‐terminal catalytic PTP domain, as the primary ubiquitination site. TRIM21 promotes K48‐linked polyubiquitination at K956, thereby regulating PTPN14 protein stability through proteasomal degradation. Given that K956 resides within the PTP catalytic domain, it remains an open question whether ubiquitination at this site also impairs phosphatase activity, in addition to reducing protein abundance. Thus, PTPN14 signaling may potentially be suppressed through dual mechanisms involving both decreased protein stability and direct inhibition of enzymatic activity, an issue that warrants further investigation. Notably, UBR4 has also been reported to be required for HPV‐16 E7‐induced degradation of PTPN14 [40]. However, our proteomic analyses did not reveal interaction between PTPN14 and UBR4, indicating TRIM21 might be the principal E3 ubiquitin ligase mediating PTPN14 ubiquitination in ECs. This discrepancy may be attributable to tissue‐specific expression patterns, as UBR4 is predominantly expressed in endoderm‐derived cells but is largely absent from mesoderm‐derived vascular tissues [41]. In addition to conventional K48‐ and K63‐linked ubiquitination, TRIM21 can also mediate atypical ubiquitination of its substrates. For instance, recent studies in tumor cells have demonstrated that TRIM21 mediates the K27‐linked ubiquitination of AKT [42] and the K11‐linked ubiquitination of ID1 (inhibitor of DNA binding 1) [43]. Hence, whether TRIM21 concurrently mediates other atypical forms of ubiquitination on PTPN14, thereby modulating its function, warrants further investigation.

In addition to TRIM21, our LC‐MS/MS analysis indicated that TRIM25 interacts with PTPN14 in ECs (Figure S3A). Previous studies showed that Global TRIM25 deficiency on an ApoE^−/−^ background developed smaller atherosclerotic plaques and exhibited attenuated vascular inflammation in mice [44], highlighting a critical role for TRIM25 in atherosclerosis. However, in contrast to the pro‐inflammatory effects of TRIM21 under disturbed flow conditions, TRIM25 has been reported to promote STAT1 degradation, thereby exerting an anti‐inflammatory effect during EC activation [45]. Given that both TRIM25 and TRIM21 possess a C‐terminal SPRY domain, which mediates the interaction between TRIM21 and PTPN14, TRIM25 might similarly bind to PTPN14 and affect its ubiquitination status. Furthermore, deubiquitinating enzymes (DUBs) counter‐regulate E3 ligase activity to finely tune protein ubiquitination levels. Our LC‐MS/MS analysis identified USP10 as the top‐scoring DUB candidate, which might interact with PTPN14 in ECs (Figure S3A). USP10 has previously been shown to bind the NOTCH1 intracellular domain (NICD1), thereby stabilizing activated NOTCH1 and suppressing angiogenic sprouting [46], indicating a critical role in ECs. However, whether DUBs such as USP10 could counterbalance TRIM21‐mediated ubiquitination of PTPN14 in ECs remains unknown. Collectively, our current findings demonstrated that TRIM21‐mediated PTPN14 degradation represents a critical molecular mechanism underlying atherosclerosis.

TRIM21 is a member of the TRIM family of E3 ubiquitin ligases and has been implicated in biological processes including innate immune signaling, tumorigenesis, and autophagy [47]. In the cardiovascular system, global ablation of TRIM21 has been shown to protect against doxorubicin‐induced cardiotoxicity and to attenuate myocardial infarction‐induced atrial fibrosis in mice, highlighting an important role for TRIM21 in cardiac remodeling [48, 49]. However, the contribution of TRIM21 to vascular pathology has remained largely unexplored. In the present study, we found that TRIM21 protein expression is not altered by distinct flow patterns, consistent with a previous report showing minimal changes in TRIM21 abundance under oscillatory vs. laminar shear stress [50]. Despite its stable expression, we identify TRIM21 as a key E3 ubiquitin ligase that mediates PTPN14 ubiquitination and proteasomal degradation in ECs, a mechanism that deserves further verification in a purified in vitro system. Notably, we generated EC‐specific TRIM21 knockout mice and demonstrated that loss of TRIM21 suppresses endothelial inflammatory activation and slows the development of atherosclerotic lesions. Interestingly, we observed a significant increase in collagen deposition in the aortic roots of *Ldlr^−/−^TRIM21^iECKO^* mice, whereas no such alterations were detected in *ApoE^−/−^PTPN14^iECKO^* mice compared to their respective littermate controls. While distinct genetic backgrounds of the respective mouse models might contribute to this divergence, this phenotypic discrepancy indicates that TRIM21 might exert multifaceted functions beyond merely mediating PTPN14 degradation. Consistent with this concept, TRIM21 could bind and ubiquitinate SOCS3, thereby enhancing NLRP3 inflammasome‐mediated pyroptosis [51]. Besides, under disturbed shear stress conditions, increased interaction between TRIM21 and MAPK6 in HUVECs has been reported to promote MAPK6 ubiquitination and proteasomal degradation, which in turn exacerbates endothelial inflammation through activation of the EGR1/NF‐κB/CXCL12 axis [50]. Intriguingly, in that study, a reduction in MAPK6 levels was detectable as early as 30 min postdisturbed flow exposure [50], suggesting that TRIM21‐mediated degradation of MAPK6 is a rapid, acute cellular event. In contrast, our findings reveal that the OSS‐induced increase in TRIM21 condensates does not manifest until 6 h of stimulation and persists up to 12 h. This temporal divergence implies that while TRIM21‐mediated degradation processes could occur acutely, TRIM21 LLPS represents a chronic or sustained adaptation to prolonged disturbed flow conditions. Together with our data, these observations support the notion that TRIM21 functions as a critical E3 ubiquitin ligase governing endothelial inflammatory responses, and that targeted inhibition of its E3 ligase activity may represent a promising therapeutic strategy for atherosclerosis. Mechanistically, our study reveals a previously unrecognized role of LLPS in TRIM21‐mediated regulation of PTPN14 under distinct flow conditions. While a previous study reported that TRIM21 could undergo LLPS in an in vitro system [52], we demonstrated that LLPS process of TRIM21 in ECs is dynamically regulated by shear stress patterns. Compared with PSS, OSS markedly enhances TRIM21 phase separation, as evidenced by increased punctate, droplet‐like structures. Importantly, TRIM21 phase separation is essential for efficient PTPN14 degradation, likely by locally increasing TRIM21 concentration and thereby enhancing ubiquitination efficiency. These findings uncover a novel mechanistic paradigm in which TRIM21 phase separation enables ECs to decode hemodynamic cues and selectively regulate protein turnover. Consistent with this concept, emerging evidence indicates that LLPS in ECs is tightly coupled to shear stress signaling. For instance, OSS has been shown to impair the phase separation capacity of Kindlin‐2, resulting in increased endothelial permeability [27]. Our data further suggest that flow‐dependent cytoskeletal remodeling may underlie changes in TRIM21 phase behavior. Previous studies have shown that TRIM21 can associate with F‐actin [29], raising the possibility that the aligned actin cytoskeleton induced by PSS inhibits TRIM21 release and subsequent phase separation. Given that emerging evidence indicates that LLPS is dynamically regulated by various post‐translational modifications (PTMs) that modulate protein multivalency, solubility, and electrostatic interactions [53], whether specific PTMs of TRIM21 contribute to its phase separation or its subsequent interaction with PTPN14 remains an intriguing open question. Furthermore, how distinct phase‐separating proteins respond to hemodynamic forces and coordinate endothelial function under distinct flow conditions remains an important area for future investigation.

As a limitation, all animal experiments in the current study were conducted exclusively using male mice to minimize estrogen‐mediated confounding variations in plaque development. Consequently, whether the mechanosensitive TRIM21‐PTPN14 cascade exhibits sex‐specific dimorphism in the female vascular endothelium remains to be elucidated.

In conclusion, our study identifies endothelial PTPN14 as a previously unrecognized mechanosensor that integrates hemodynamic cues to regulate endothelial inflammatory activation and atherogenesis. We identify TRIM21 as a critical E3 ubiquitin ligase that governs PTPN14 protein stability through K48‐linked ubiquitination at K956, a process that is selectively enhanced under disturbed flow conditions via TRIM21 LLPS. These findings highlight phase separation‐dependent ubiquitin signaling as a fundamental layer of vascular mechanotransduction and suggest that targeting TRIM21‐mediated PTPN14 degradation may represent a promising therapeutic strategy for the prevention and treatment of atherosclerosis.

## 4 Experimental Section

The complete and detailed materials and methods are provided in the Supporting Information.

### 4.1 Ethical Approval

All study protocols for animal experiments were approved by the Institutional Animal Care and Use Committee of Tianjin Medical University and conformed to the current NIH guidelines for Care and Use of Laboratory Animals. Written informed consent was obtained from all participants.

### 4.2 Cell Culture, Transfection, and Shear Stress Experiments

HUVECs were obtained and cultured as previously described [54], derived from normal pregnancies; cells were obtained following written informed consent from donors. The study protocol was approved by the Ethics Committee of Experimental Research at Tianjin Medical University General Hospital. Experimental procedures using human tissues conformed to the principles outlined in the Declaration of Helsinki and were performed with the approval of Tianjin Medical University and Ethics Committees. Cells were incubated at 37°C in a humidified environment containing 5% CO_2_ and grown to 70%‐80% confluence before treatment. For flow experiments, confluent monolayers of HUVECs were seeded onto glass slides, and a parallel plate flow system was used to launch oscillatory flow (0.5 ± 4 dyn/cm^2^, 1 hz) and pulsatile flow (12 ± 4 dyn/cm^2^, 1 hz). The flow system was enclosed in a chamber. MG132 (TargetMol, T2514, 10 µM) in DMSO, CHX (Adooq Bioscience, A10036, 50 µg/mL) in DMSO, and CQ (TargetMol, T8689, 10 µM) in DMSO were added to the system for 6 h. For live cell imaging, 1,6‐HD (Macklin, H810887) and 2,5‐HD (Macklin, H823950) were supplemented into the medium. Primary bovine aortic ECs (BAECs) and HEK293T cells were cultured with DMEM supplemented with 10% FBS. THP‐1 cells (Cat NO. TIB‐202, ATCC) were cultured with RPMI 1640 medium with 10% FBS. All cells and the flow system were maintained at 37°C in a humidified atmosphere containing 5% CO_2_.

### 4.3 Animals

Mice were anesthetized by using isoflurane (2%‐3%). In all experiments, mice were euthanized by cervical dislocation under isoflurane inhalation. All animal care and experimental procedures conformed to the Guide for the Care and Use of Laboratory Animals by the US National Institutes of Health (NIH Publication No. 85‐23, revised in 2011) and were approved by the Institutional Animal Care and Use Committee of Tianjin Medical University. Apolipoprotein E‐null (*ApoE^−/−^*) and LDL receptor‐null (*Ldlr^−/−^*) mice were purchased from the Experimental Animal Centre of Military Medical Science Academy (Beijing, China). The *PTPN14^flox^* mice (Strain NO. T020775) and *TRIM21^flox^* mice (Strain NO. T051862) were generated from GemPharmatech (Nanjing, China). Briefly, *PTPN14^flox^* and *TRIM21^flox^* transgenic mice were crossbred with *Cdh5‐Cre* mice to generate *PTPN14^iECKO^*, *TRIM21^iECKO^* and *PTPN14^iECKO^TRIM21^iECKO^* mice. *PTPN14^iECKO^* and *TRIM21^iECKO^* mice respectively crossbred with *ApoE^−/−^* and *Ldlr^−/−^* mice to generate *PTPN14^iECKO^ApoE^−/−^* and *TRIM21^iECKO^Ldlr^−/−^* mice. For experiments using AAV‐mPCSK9^D377Y^, a total volume of 200 µL containing 1 × 10^11^ vector genomes(vg)/mouse, were injected into the tail veins of *PTPN14^iECKO^* and *PTPN14^iECKO^TRIM21^iECKO^* mice. The 8‐week‐old male mice were used in all in vivo studies. The animals were kept at a constant temperature (21 ± 1°C) under a 12/12‐h light/dark cycle and had free access to water and standard chow unless otherwise specified. Mice were fed with a high‐fat diet (Research Diets, Cat No. D12108C) for 12 weeks. For all animal experiments, male mice were exclusively used to minimize estrogen‐mediated confounding variations in plaque development.

### 4.4 Carotid Artery Partial Ligation

Mice were anesthetized with isoflurane (2%–3%). A ventral midline incision (4–5 mm) was made in the neck, and the left carotid artery was exposed through blunt dissection of subcutaneous fat and muscle tissue. The left external carotid, internal carotid, and occipital arteries were ligated with a 6‐0 silk suture, leaving the superior thyroid artery intact. The adenovirus (Ad‐Myc‐TRIM21 or Ad‐NC) was introduced into the lumen of the left carotid artery and kept inside for 30 min. After infection, the adenovirus was released, and blood flow to the common carotid artery was restored. At the end of the experiments, mice were anaesthetized by inhalation of 2% isoflurane and then killed by cervical dislocation. Mice were fed with a high‐fat diet (Research Diets, Cat No. D12108C) immediately after surgery and continued for 2 weeks.

### 4.5 Human Samples

Human coronary arteries samples were obtained as we previously reported [55]. Studies using autopsy tissue were adhered to the declaration of Helsinki and approved by the Ethics Committee of Shantou University Medical College (SUMC‐2025‐039) and family members of subjects provided written informed consent for autopsy. The arterial samples were divided into non‐atherosclerotic and atherosclerotic arteries based on their morphology. HE and immunofluorescence staining were performed as described above.

### 4.6 Statistical Analysis

The statistical data were presented as means ± SEM. The data were tested for normality before parametric statistics using the Shapiro‐Wilk normality test. For normally distributed data, we conducted comparisons between two groups using the unpaired Student's *t* test, whereas three or more groups were analyzed using the one‐way ANOVA followed by the Tukey's multiple comparisons test. For non‐normal data, we utilized the Mann‐Whitney *U* test or the Kruskal‐Wallis test followed by Dunn's multiple comparison test. Furthermore, for data with two or more subcategories, we employed a two‐way ANOVA followed by Tukey's multiple comparison test. All statistical analyses were performed with Graphpad Prism 8.0 software. *p* value<0.05 was considered statistically significant.

## Author Contributions

X.H., Q.M., Y.C., Q.B., Y.Z., and J.H. designed the study. X.H. and Q.M. performed experiments and statistical analysis. X.W., J.M., and B.W. helped with the animal study. C.L. performed molecular docking experiments. L.Y., and Y.C. designed research studies with human samples and helped revise the manuscript. The manuscript was written by X.H., Y.Z., and J.H. All authors reviewed the paper and approved the final version.

## Funding

This work was supported by the National Natural Science Foundation of China (82330012, 82422006, 82127808, 32471166, 82570543, 82270516, 82370258, 82400549), Natural Science Foundation of Tianjin (24JCJQJC00060 and 24JCYBJC01140), Tianjin Municipal Education Commission (2025ZD007 and 2025ZD030), and Discipline Research Special Project of Tianjin Medical University (2024XKNFM09).

## Conflicts of Interest

The authors declare no conflicts of interest.

## Supporting information




**Supporting File**: advs77262‐sup‐0001‐SuppMat.docx.

## Data Availability

The data that support the findings of this study are available on request from the corresponding author. The data are not publicly available due to privacy or ethical restrictions.
