# Supplementary material for: Phase Separation of TRIM21 Modulates PTPN14 Stability to Drive Flow‐Dependent Endothelial Activation and Atherogenesis
Source: Adv Sci (Weinh). 2026 Aug 18:e77262. Online ahead of print. doi: 10.1002/advs.77262 (PMC13482943; doi:10.1002/advs.77262)
Supplement: Supplementary file 1 [file ADVS-9999-e77262-s001.docx]

**Supporting Information**

Figures S1-8

Tables S1-4

**Title**

**Phase separation of TRIM21 modulates PTPN14 stability to drive flow-dependent endothelial activation and atherogenesis**

*Xue He^1,2,3#^, Qiannan Ma^1,2#^, Xue Wang^1,2^, Jiaxu Mang^2^, Chufeng Li^1,2^, Bingjun Wu^1,2^, Liu Yao^2^, Yequn Chen^3*^, Qiankun Bao^4*^, Yi Zhu^2*^, Jinlong He^1,2*^*

^1^Province and Ministry Co-sponsored Collaborative Innovation Center for Medical Epigenetics, Eye Institute and School of Optometry, Tianjin Medical University Eye Hospital, Tianjin Medical University, Tianjin, 300070, China;

^2^NHC Key Laboratory of Hormones and Development, Department of Physiology and Pathophysiology, Tianjin Medical University, Tianjin, 300070, China;

^3^Department of Cardiology, First Affiliated Hospital of Shantou University Medical College, Guangdong, 515041, China;

^4^Tianjin Key Laboratory of Ionic-Molecular Function of Cardiovascular Disease, Department of Cardiology, Tianjin Institute of Cardiology, the Second Hospital of Tianjin Medical University, Tianjin, 300211, China.

*Xue He* and *Qiannan Ma* contributed equally to this work.

***Correspondence:**

Jinlong He, Ph.D.

Department of Physiology and Pathophysiology, Tianjin Medical University; Qixiangtai Rd 22^nd^, Tianjin, 300070, China.

Tel: +86-22-83336591; E-mail: hejinlong@tmu.edu.cn

or

Yi Zhu, MD.

Department of Physiology and Pathophysiology, Tianjin Medical University; Qixiangtai Rd 22^nd^, Tianjin, 300070, China.

Tel: +86-22-83336591; E-mail: zhuyi@tmu.edu.cn

or

Qiankun Bao, MD.

Tianjin Institute of Cardiology, the Second Hospital of Tianjin Medical University, 300211, Tianjin, China.

Tel: +86-22-88326237; E-mail: baoqiankun@tmu.edu.cn

or

Yequn Chen, MD.

Department of Cardiology, First Affiliated Hospital of Shantou University Medical College, Guangdong, 515041, China.

E-mail: gdcycyq@163.com

**Supplementary Methods**

**Lipid measurements**

Plasma was separated by centrifugation. Total plasma cholesterol (CHO), triglycerides (TGs), low-density lipoprotein cholesterol (LDL-C), and high-density lipoprotein cholesterol (HDL-C) levels were enzymatically measured by use of an automated clinical chemistry analyzer kit (Biosino Biotech, Beijing, China).

**Histological analysis of atherosclerotic lesion**

Aortic trees were fixed with 4% paraformaldehyde and then stained with Oil Red O to quantify the lesion area by using Image-Pro Plus (Media Cybernetics, Rockville, MD, USA). The cross-sections of aortic roots were fixed in 4% paraformaldehyde and embedded in optimal cutting temperature compound (OCT). OCT-embedded tissues were sectioned at a thickness of 7 μm. The cross sections of aortic roots were stained with Oil Red O, hematoxylin and eosin (HE), and Masson to assess lipid accumulation, lesion area, and collagen deposition.

**En face analysis of aortic endothelium**

Mice were anesthetized and fixed with 4% paraformaldehyde in PBS buffer for 10 min by perfusion through left cardiac ventricle under physiological pressure. Aortas were harvested, further fixed with 4% paraformaldehyde in PBS buffer for 30 min and were then longitudinally dissected with micro dissecting scissors. The luminal surfaces of the aortas were exposed, blocked with 3% bovine serum albumin in PBS for 30 min at room temperature, and were incubated with primary antibodies at 4 °C overnight. The PTPN14 antibody (Cat NO. sc-373766) was purchased from Santa Cruz Biotechnology. The TRIM21 antibody (Cat NO. 12108-1-AP) was from Proteintech. The VE-cadherin antibody (Cat. No. ab33168) were obtained from Abcam (Cambridge, UK). Following primary antibody incubation, the aortas were washed three times with PBS and were then probed with secondary antibodies including Alexa Fluor 488-conjugated goat anti-rabbit IgG (1:200) or Alexa Fluor 594-conjugated goat anti-mouse IgG (1:200). Nuclei were counterstained with DAPI for 5 min at room temperature. Image acquisition was performed using a laser-scanning confocal microscopy (LSM900, CarlZeiss).

**Immunofluorescence staining**

The cross sections of aortic roots were washed with PBS for 5 min and then fixed with 4% paraformaldehyde for 15 min. And permeabilized in 0.1% Triton X-100 (in PBS) and blocked with 1% bovine serum albumin for 30 min at room temperature. Then primary antibodies (1:100) were incubated overnight at 4°C. The CD68 (Cat. No. ab955) vWF (Cat. No. ab11713), and p-YAP^Y357^(Cat. No. ab 62751) antibodies were obtained from Abcam (Cambridge, UK). VCAM-1(Cat. No. sc-13160) antibody was from Santa Cruz Biotechnology (Santa Cruz, CA, USA). The α-SMA antibody (Cat No. 19245), p-YAP^S127^(Cat No. D9W2I) was purchased from Cell Signaling Technology (Danvers, MA, USA). Following primary antibody incubation, sections were treated with Alexa Fluor 488- or Alexa Fluor 594-conjugated secondary antibodies (1:200, Thermo Fisher Scientific, Grand Island, NY, USA) at room temperature for 1 hr. Cross mounted with DAPI-containing mounting medium. All immunofluorescence micrographs were acquired by laser-scanning confocal microscopy (LSM900, CarlZeiss).

**Recombinant adenovirus construction and transfection**

The adenovirus expressing Myc-tagged human TRIM21 (NM_003141.4, Ad-TRIM21), Flag-tagged human PTPN14 (NM­­_005401.5, Ad-PTPN14), and control empty vector (Ad-NC) were constructed in GeneChem (Shanghai, China). HUVECs were infected with adenovirus at multiplicity of infection (MOI) 1 for 48 h.

**Small interfering RNAs (siRNAs) and plasmids transfection**

SiRNA targeting TRIM21 was from Sangon Biotech (Shanghai, China). The sequences of siRNA were listed in Table S1. HUVECs were pre-incubated in the transfection medium (Opti-MEM, Cat NO. 31985070, Thermo Fisher Scientific) and then transiently transfected with siRNAs by Lipofectamine™ RNAiMAX (Cat NO. 13778150, Thermo Fisher Scientific).

The plasmids of full-length PTPN14, PTPN14 mutants, full-length TRIM21, TRIM21 mutants, PTPN14 and TRIM21 domain truncations were constructed by GENEWIZ (Suzhou, China). These plasmids were transfected into HEK293T cells by using Lipofectamine3000 (Cat NO. L3000-015, Thermo Fisher Scientific) for 48 h.

**Western blots analysis**

HUVECs and aortic tissues were lysed in ice-cold RIPA buffer (Cat NO. P0013B, Beyotime Biotechnology, Beijing, China) containing 1% PMSF (Cat NO. P0100, Solarbio Life Sciences, Beijing, China), 1x protease inhibitor cocktail (Cat NO. 04693132001, Roche, Indianapolis, IN, USA) and phosphatase inhibitor (Cat NO. 04906845001, Roche). The concentration of supernatants was measured with BCA protein assay (Cat NO. 23227, Thermo Fisher Scientific). Protein extracts were subjected to SDS-PAGE, transferred to nitrocellulose membranes (Cytiva). Analysis involved the primary antibodies for Ubiquitin (Cat NO. 3936, Cell Signaling Technology), K48-Ubiquitin (Cat NO. 8081, Cell Signaling Technology), TRIM21 (Cat NO. 92043, Cell Signaling Technology), Flag (Cat NO. 14793, Cell Signaling Technology), Myc (Cat NO. 2276, Cell Signaling Technology), HA (Cat NO. 3724, Cell Signaling Technology), β-actin (Cat NO. 3700, Cell Signaling Technology), t-eNOS (Cat NO. 32027, Cell Signaling Technology), PTPN14 (Cat NO. sc-373766, Santa Cruz Biotechnology), VCAM-1(Cat. No. sc-13160, Santa Cruz Biotechnology), and GAPDH (Cat NO. 60004-1-Ig, Proteintech). Proteins were visualized with HRP-conjugated anti-rabbit or anti-mouse IgG (1:2000 or 1:8000; Cell Signaling Technology), followed by use of the ECL chemiluminescence system (Cell Signaling Technology). The images were analyzed by using Image J v1.52.

**Monocyte adhesion**

THP-1 cells were labelled with BCECF-AM (Thermo Fisher Scientific, Grand Island, NY, USA) and then plated onto the HUVECs glass slides that underwent shear stress at 2 × 10^6^ cells per well. After incubation for 60 min at 37°C, non-adherent cells were removed by washing three times with PBS. In each sample, the number of stained adhering cells in five random fields was counted under a fluorescence microscope.

**Quantitative real-time PCR**

Total RNA was extracted by using a kit (Cat NO.5105, NCM biotech, Suzhou, China). Reverse transcription involved use of a reverse transcription kit (NCMScript All-in-One RTpremix with dsDNase, NCM biotech, Suzhou, China). Quantitative real-time PCR (qRT-PCR) was performed with SYBR Green PCR Master Mix (Cat NO. M7205, NCM biotech, Suzhou, China). Gene expression was normalized to that of GAPDH. The gene-specific primer sequences are listed in Table S2.

**Immunoprecipitation**

Cells were lysed in IP lysis (50 mM Tris-HCl, 150 mM NaCl, 1% Nonidet-P40) containing 1% PMSF, 1x protease inhibitor cocktail (Cat NO. 04693132001, Roche), and phosphatase inhibitor (Cat NO. 04906845001, Roche). For endogenous IP experiments, the cell lysates were incubated with 1- to 2-μg specific antibody overnight at 4°C with constant rotation. The lysates incubated with IgG antibody were served as negative controls. Protein A/G PLUS-Agarose beads (Cat NO. 20423, Thermo Fisher Scientific) were added in the lysates for reaction at room temperature for 2 h. After a final wash, the supernatant was discarded, and the precipitated proteins were eluted from the beads by resuspending in 2× SDS-PAGE loading buffer and boiling for 5 min. For immunoprecipitation with HEK293T cells, the cells lysates were immunoprecipitated with anti-Flag (Cat NO. B26101, Bimake, Shanghai, China) or anti-Myc (Cat NO. B26301, Bimake) affinity beads at 4°C overnight. The resultant materials from immunoprecipitation or cell lysates underwent western blot analysis.

**Proximity Ligation Assay**

HUVECs after fixed with 4% paraformaldehyde and permeabilized with 0.1% Triton X-100 for 30 min, then blocked with goat serum for 30 min following by washing with wash buffer. Incubation with primary antibodies against PTPN14 (Cat NO. sc-373766, Santa Cruz Biotechnology), TRIM21 (Cat NO. 12108-1-AP, Proteintech) at 4°C overnight. The dilution ratio used for all antibodies was 1:200. Subsequently, the staining was performed by using the Rabbit PLUS and Mouse MINUS Duolink in situ proximity ligation assay (PLA) kits (Cat NO. DUO92101, Sigma-Aldrich) according to the manufacturer’s protocol. The nuclei were counterstained with DAPI. Immunofluorescent signals were captured by Zeiss (LSM900) confocal laser scanning microscopy.

**Supplemental figures and figure legends**

**Figure S1. (A)** PTPN14 mRNA expression in intima from the thoracic aorta (TA) and aortic arch (AA) of ApoE^-/-^ mice. Aortic intima from 2 mice were pooled as 1 sample. Data are mean ± SEM, n = 3 pooled samples. **(B)** Quantification of PTPN14 mRNA expression in HUVECs under OSS (0.5 ± 4 dyn/cm^2^, 1 Hz) or PSS (12 ± 4 dyn/cm^2^, 1 Hz) for 6 h. Data are mean ± SEM, n = 6. **(C-D)** HUVECs were exposed to oscillatory shear stress (OSS; 0.5 ± 4 dyn/cm², 1 Hz) or pulsatile shear stress (PSS; 12 ± 4 dyn/cm², 1 Hz) in the presence or absence of MG132 (10 μM) or CQ (10 μM) for 6 h, followed by immunoblot analysis of PTPN14 proteins levels. Data are mean ± SEM, **P*<0.05 (two-way ANOVA with Tukey’s multiple comparison post-hoc test), n = 5.

**Figure S2. (A)** Genotyping PCR for *Ptpn14^fl/fl^* and *Ptpn14^iECKO^* mice. **(B)** Representative en face immunofluorescence staining of PTPN14, VE-cadherin, and DAPI, and quantification of PTPN14 expression. Scale bar, 10 μm. **(C)** The immunofluorescence intensity of PTPN14 was normalized to that of DAPI, and the relative expression values were compared to that of the *Ptpn14^fl/fl^*. Data are mean ± SEM, **P*<0.05 (2-tailed unpaired Student’s *t* test), n = 6 mice per group. **(D)** Representative immunoblot images of PTPN14 in lung, kidney, and liver tissues isolated from *Ptpn14^fl/fl^* and *Ptpn14^iECKO^* mice. **(E)** Quantification of PTPN14 protein levels in the corresponding tissues. Data are mean ± SEM, n = 6 mice. **(F)** Quantification of body weight at the beginning and the end of experiment. Data are mean ± SEM; n = 10 mice per group. **(G)** Quantification of plasma levels of total cholesterol (CHO), triglycerides (TG), high-density lipoprotein cholesterol (HDL-C) and low-density lipoprotein cholesterol (LDL-C). Data are mean ± SEM; n = 10 mice per group.

**Figure S3. (A)** Mass spectrometry-based proteomic screening identifying candidate E3 ubiquitin ligases (E3s) and deubiquitinating enzymes (DUBs) associated with PTPN14. **(B-C)** HUVECs were transfected with negative control siRNA (siNC) or TRIM21 siRNA (siTRIM21-2#) for 36 h, then exposed to oscillatory shear stress (OSS) or pulsatile shear stress (PSS) in the presence of cycloheximide (CHX; 50 μg/mL) for 6 h. Representative immunoblots of PTPN14 are shown. Data are mean ± SEM, **P*<0.05 (two-way ANOVA with Tukey’s multiple comparison post-hoc test), n = 6 independent experiments.

**Figure S4. (A)** HEK293T cells were cotransfected with Myc-tagged empty vector or Myc-TRIM21 together with Flag-tagged PTPN14 and HA-tagged ubiquitin (HA-Ub; wild-type, K63-only, or K63R) for 36 h. Cells were then treated with MG132 (10 μM) for 6 h before lysis. PTPN14 was immunoprecipitated from cell lysates, followed by immunoblotting as indicated. n = 3 independent experiments.

**Figure S5. (A-B)** HUVECs were subjected to OSS (0.5 ± 4 dyn/cm^2^, 1 Hz) or PSS (12 ± 4 dyn/cm^2^, 1 Hz) for 12 h. Immunoblotting was performed to assess TRIM21 protein expression, with quantification shown. Data are mean ± SEM (n = 6 independent experiments). **(C-D)** Representative en face immunofluorescence images (C) of TRIM21, VE-cadherin, and DAPI, and quantification (D) of condensate number in the AA and TA of mice. Scale bar, 5 µm. Data are mean ± SEM (n = 6 mice per group), **P*<0.05 (2-tailed unpaired Student’s *t* test), **(E-F)** Bovine aortic endothelial cells (BAECs) were transfected with Myc-tagged wild-type TRIM21 or a coiled-coil–deleted mutant (TRIM21-ΔCC). Representative immunofluorescence images and quantification of condensate number per cell are shown. Scale bar, 20 µm. Data are mean ± SEM, **P*<0.05 (two-way ANOVA with Tukey’s multiple comparison post-hoc test), n = 6 independent experiments.

**Figure S6. (A)** HUVECs were infected with control adenovirus (Ad-NC) or Ad-Myc-TRIM21 at increasing multiplicities of infection (MOI: 0.1, 1, 10, and 100) for 36 h. Representative immunoblots of Myc-TRIM21 are shown. **(B-D)** HUVECs were transduced with control adenovirus (Ad-NC) or TRIM21 adenovirus (Ad-TRIM21) at an MOI of 1 for 36 h and then exposed to OSS (0.5 ± 4 dyn/cm^2^, 1 Hz) or PSS (12 ± 4 dyn/cm^2^, 1 Hz) for 6 h. **(B)** Reverse transcription-quantitative PCR analysis of VCAM-1 and ICAM-1 mRNA expressions. Data are mean ± SEM, **P*<0.05 (two-way ANOVA with Tukey’s multiple comparison post-hoc test), n = 5 independent experiments. **(C-D)** THP-1 monocytes were pre-labeled and co-cultured with HUVECs for 30 min. Representative images and quantification of adherent THP-1 cells are shown. Scale bar, 200 µm. Data are mean ± SEM, **P*<0.05 (two-way ANOVA with Tukey’s multiple comparison post-hoc test), n = 6 independent experiments. **(E-K)** *Ldlr^-/-^* mice underwent partial ligation of the left common carotid artery and were locally incubated with control adenovirus (Ad-NC) or Ad-TRIM21. After surgery, the mice fed with high fat diet for 2 weeks. **(E)** Representative images of En face immunofluorescence staining of TRIM21 and VE-cadherin in the left common carotid artery, with nuclei counterstained with DAPI. Scale bar, 10 μm. **(F)** Representative images of aortic arches and hematoxylin and eosin (HE) staining of carotid artery cross-sections. Scale bar, 50 µm. **(G)** Lesion area in carotid arteries was quantified. Data are mean ± SEM, **P*<0.05 (2-tailed unpaired Student’s *t* test), n = 10 mice per group. **(H-K)** Representative immunofluorescence staining and quantification of PTPN14 and vascular cell adhesion molecule-1 (VCAM-1) in endothelium of left carotid arteries. Scale bar, 50 µm (H) and 100 µm (J), Data are mean ± SEM, **P*<0.05 (2-tailed unpaired Student’s *t* test), n = 10 mice per group.

**Figure S7. (A)** Genotyping PCR for *Trim21^fl/fl^* and *Trim21^iECKO^* mice. **(B-C)** Representative en face immunofluorescence staining of TRIM21, VE-cadherin, and DAPI, and quantification of TRIM21 expression. Scale bar, 10 μm. Data are mean ± SEM, **P*<0.05 (2-tailed unpaired Student’s *t* test), n = 6 mice per group. **(D)** Representative immunofluorescence images of PTPN14 and von Willebrand factor (vWF) in aortic root sections, with nuclei counterstained with DAPI. Scale bar, 20 µm. **(E**) Quantification of intimal PTPN14 fluorescence intensity (normalized to DAPI). Data are mean ± SEM, **P*<0.05 (2-tailed unpaired Student’s *t* test), n = 10 mice per group. **(F)** Quantification of body weight at the beginning and the end of experiment. Data are mean ± SEM, n = 10 mice per group. **(G)** Quantification of plasma levels of total cholesterol (CHO), triglycerides (TG), high-density lipoprotein cholesterol (HDL-C) and low-density lipoprotein cholesterol (LDL-C). Data are mean ± SEM, n = 10 mice per group, except for the HDL-C analysis, for which n = 9 mice per group.

**Figure S8. (A)** Quantification of body weight at the beginning and the end of experiment. Data are mean ± SEM, n = 6 mice per group. **(B)** Quantification of plasma levels of total cholesterol (CHO), triglycerides (TG), high-density lipoprotein cholesterol (HDL-C) and low-density lipoprotein cholesterol (LDL-C). Data are mean ± SEM, n = 6 mice per group, except for the *Ptpn14^iECKO^Trim21^iECKO^* group in the LDL-C analysis, for which n = 5 mice.

**Supplemental Tables**

**Table S1.** **The sequences of TRIM21 siRNA.**

| **Gene** | **Strand** | **Sequence** |
| --- | --- | --- |
| siTRIM21 | Sense | GAGUUGGCUGAGAAGUUGGAATT |
|  | Antisense | UUCCAACUUCUCAGCCAACUCTT |
| siTRIM21-2# | Sense | GAAGAGAGAUUUGAUAGUUAUTT |
|  | Antisense | AUAACUAUCAAAUCUCUCUUCTT |

**Table S2.** **The sequences of qRT-PCR primers**.

| **No.** | **Gene** | **Strand** | **Sequence** |
| --- | --- | --- | --- |
| 1 | Human PTPN14 | Sense | TGTGGGAGCAGGGAGTGAATG |
|  |  | Antisense | AACTTCGTGGTGACCTTGAACTTG |
| 2 | Human GAPDH | Sense | AAGACGGGCGGAGAGAAACC |
|  |  | Antisense | CGTTGACTCCGACCTTCACC |
| 3 | Human VCAM-1 | Sense | CGAACCCAAACAAAGGCAGA |
|  |  | Antisense | ACAGGATTTTCGGAGCAGGA |
| 4 | Human ICAM-1 | Sense | TTGGGCATAGAGACCCCGTT |
|  |  | Antisense | GCACATTGCTCAGTTCATACACC |
| 5 | Human SELE | Sense | GCCTGCAATGTGGTTGAGTG |
|  |  | Antisense | GCACCTCACAGAGCCATTCT |
| 6 | Human MCP-1 | Sense | TTTCCCCTAGCTTTCCCCAG |
|  |  | Antisense | ACCCTCAAAACATCCCAGGG |
| 7 | Mouse PTPN14 | Sense | GCAAGCTGACTTCGGAGATT |
|  |  | Antisense | AGGATCCCGCTATGAGCTTT |
| 8 | Mouse GAPDH | Sense | CACGGCAAATTCAACGGCACAG |
|  |  | Antisense | TCGCTCCTGGAAGATGGTGATGG |

**Table S3. The ubiquitination sites of PTPN14 predicted by PhosphoSitePlus.**

| **No.** | **Position** | **Sequence** |
| --- | --- | --- |
| 1 | k6 | MPFGLkLRRTRRy |
| 2 | k18 | RRyNVLSkNCFVtRI |
| 3 | k274 | ILVELINKEETALFH |
| 4 | k289 | TDDIENAkYISRLFA |
| 5 | k509 | PQGVySNkLVsPSDQ |
| 6 | k527 | KNNVVPSkPGASAIS |
| 7 | k588 | DLAsHRHkYVsGssP |
| 8 | k601 | sPDLVtRkVQLsVkT |
| 9 | k607 | RkVQLsVkTFQEDSS |
| 10 | k747 | ALARIPNkPPPEYPG |
| 11 | k757 | PEYPGPRksVsNGAL |
| 12 | k787 | VLRHGPAkAIsMSRt |
| 13 | k817 | EPDLtSVkERVKKEP |
| 14 | k920 | yEQIPKKkANGIFST |
| 15 | k956 | RVELIPTkENNTGYI |
| 16 | k1029 | YWPKLGSkHSSATYG |
| 17 | k1037 | HSSATYGkFKVTTKF |
| 18 | k1059 | ATTGLKVkHLLSGQE |

**Table S4. The ubiquitination sites of PTPN14 predicted by qPTM.**

| **No.** | **Position** | **Sequence** |
| --- | --- | --- |
| 1 | K6 | MPFGLKLRRTRRY |
| 2 | K18 | RRYNVLSKNCFVTRI |
| 3 | K123 | YQYYLQVKKDVLEGR |
| 4 | K509 | PQGVYSNKLVSPSDQ |
| 5 | K520 | PSDQRNPKNNVVPSK |
| 6 | K956 | RVELIPTkENNTGYI |
| 7 | K1043 | GKFKVTTKFRTDSVC |
